# A Poisson hierarchical modelling approach to detecting copy number variation in sequence coverage data

**DOI:** 10.1186/1471-2164-14-128

**Published:** 2013-02-26

**Authors:** Nuno Sepúlveda, Susana G Campino, Samuel A Assefa, Colin J Sutherland, Arnab Pain5, Taane G Clark

**Affiliations:** 1 London School of Hygiene and Tropical Medicine, London, UK; 2 Center of Statistics and Applications, University of Lisbon, Lisbon, Portugal; 3 Wellcome Trust Sanger Institute, Hinxton, UK; 4 Department of Clinical Parasitology, Hospital for Tropical Diseases, London, UK; 5 King Abdullah University of Science and Technology, Thuwal, Saudi Arabia

## Abstract

**Background:**

The advent of next generation sequencing technology has accelerated efforts to map and catalogue copy number variation (CNV) in genomes of important micro-organisms for public health. A typical analysis of the sequence data involves mapping reads onto a reference genome, calculating the respective coverage, and detecting regions with too-low or too-high coverage (deletions and amplifications, respectively). Current CNV detection methods rely on statistical assumptions (*e.g.*, a Poisson model) that may not hold in general, or require fine-tuning the underlying algorithms to detect known hits. We propose a new CNV detection methodology based on two Poisson hierarchical models, the Poisson-Gamma and Poisson-Lognormal, with the advantage of being sufficiently flexible to describe different data patterns, whilst robust against deviations from the often assumed Poisson model.

**Results:**

Using sequence coverage data of 7 *Plasmodium falciparum* malaria genomes (3D7 reference strain, HB3, DD2, 7G8, GB4, OX005, and OX006), we showed that empirical coverage distributions are intrinsically asymmetric and overdispersed in relation to the Poisson model. We also demonstrated a low baseline false positive rate for the proposed methodology using 3D7 resequencing data and simulation. When applied to the non-reference isolate data, our approach detected known CNV hits, including an amplification of the PfMDR1 locus in DD2 and a large deletion in the CLAG3.2 gene in GB4, and putative novel CNV regions. When compared to the recently available FREEC and cn.MOPS approaches, our findings were more concordant with putative hits from the highest quality array data for the 7G8 and GB4 isolates.

**Conclusions:**

In summary, the proposed methodology brings an increase in flexibility, robustness, accuracy and statistical rigour to CNV detection using sequence coverage data.

## Background

Recent genome research have highlighted the role of structural variants on natural phenotypic variations with vital importance for human health [1,2]. The advent of massively parallel sequencing technologies has resulted to a drastic cost reduction per megabase of DNA sequence, and is leading to unprecedented genomic resolution and large sample size applications. In a single run, these technologies are able to generate millions of DNA fragments (reads) from a target genome, which are then mapped onto a reference genome when available or undergo *de novo* assembly. The resulting mapped data with potentially high coverage is the core of structural variant detection, and several methods have been recently proposed depending on the type of polymorphism to be identified, as recently reviewed by Medvedev et al. [3].

The present work considers the detection of copy number variations (CNVs), such as deletions and amplifications, using sequence coverage data when mapped onto a reference genome. In theory, deletions are detected in regions with extremely low coverage whereas amplifications are typically located in regions with exceptionally high coverage [3]. The common strategy to analyse sequence coverage data is to divide the reference genome into non-overlapping windows (or bins) of a given size [3-6]. Since the GC content is known to influence the resulting coverage distribution [7-9], the windows are further subdivided according to this genomic parameter and analysed separately. Finally, appropriate detection limits are calculated. In this regard, there are two main approaches to determine these thresholds. One approach is to assume a Poisson distribution for coverage when there is no copy number variation, as invoked by the EWT method [4]. In theory, this assumption entails an equality between the mean and the variance of the coverage distribution. However, there is a growing number of data sets whose variance of the coverage distribution is clearly greater than the mean coverage [4,10]. This statistical “overdispersion” implies that, at a given statistical significance level, any Poisson-based method tends to detect a higher number of CNVs in comparison to a situation where overdispersion is deemed an intrinsic property of the data, thereby increasing the false positive rate. Recently, the cn.MOPS approach has been proposed, where the analysis is performed across samples and the resulting coverage distribution of a given window (across samples) is modelled through a finite mixture of Poisson distributions [6]. However, within a window, this modelling approach reverts to the common Poisson distribution (with different parameters along the different segments comprising the genome) if there is no CNV present. Another approach assumes a proportionality between the underlying copy number and the median coverage after being adjusted for the underlying GC content, and smoothed by an appropriate segmentation/aggregation algorithm, as available in the FREEC software [5]. Notwithstanding its high computational efficiency, this software critically relies on the analyst to parameterise the underlying segmentation algorithm. If prior information is available from the samples under analysis, one can set key parameters tentatively until obtaining results in line with previous findings. This fine-tuning exercise becomes extremely time consuming in a high throughput setting, especially as expected differences in the patterns of data between samples cannot be captured by a single parameter set. Alternative methodologies are then required with the potential of being much more generalisable and applicable to a high throughput setting.

To improve current approaches for CNV detection, we propose a new methodology based on a Poisson hierarchical modelling approach. Our data analysis strategy is now outlined. First, we assume a Poisson distribution for coverage when there is no copy number variation, as previously done in EWT [4] and cn.MOPS [6]. We then extend this distribution to an overdispersion setting by allowing the respective rate parameter to vary according to a Gamma or a Lognormal distribution. The resulting models of this hierarchical structure are the Poisson-Gamma (also known as the Negative Binomial) and Poisson-Lognormal, respectively. In this way, different data patterns under the hypothesis of no CNVs can be captured due to the great flexibility of these second-level distributions. We adjust the results for the GC content as implemented elsewhere, i.e., we divide the reference genome into non-overlapping windows and analyse those with similar GC content separately. The formal CNV detection is based on highest posterior density (HPD) credible intervals associated with the posterior predictive distribution for coverage. Hence the stringency of our method is controlled by the credibility level — hereafter denoted by the parameter *γ* — specified for the analysis. The final stage of the analysis encompasses merging contiguous hits and excluding putative deletions when the corresponding coverage per nucleotide position is greater than zero.

To assess the performance of our methodology, we use 7 publicly available *Plasmodium falciparum* (*Pf*, causes malaria) data sets. The choice of this real-world data set shows two major advantages. First, it includes the re-sequencing data of the 3D7 reference genome thus allowing the direct estimation of the baseline false positive rate. We also used this reference sample to perform a simulation study where data shows different read depth. Second, it encompasses data of 4 laboratory strains for which comparative genomic hybridization (CGH) array data is available, thus enabling ’external’ validation of the CNVs detected via sequencing data analysis. All results are compared to the FREEC and cn.MOPS softwares, two potentially promising approaches when applied to human genome data [6].

## Results

### *Plasmodium falciparum* genome data shows distinct random patterns for the underlying coverage distribution

**Figure 1:**
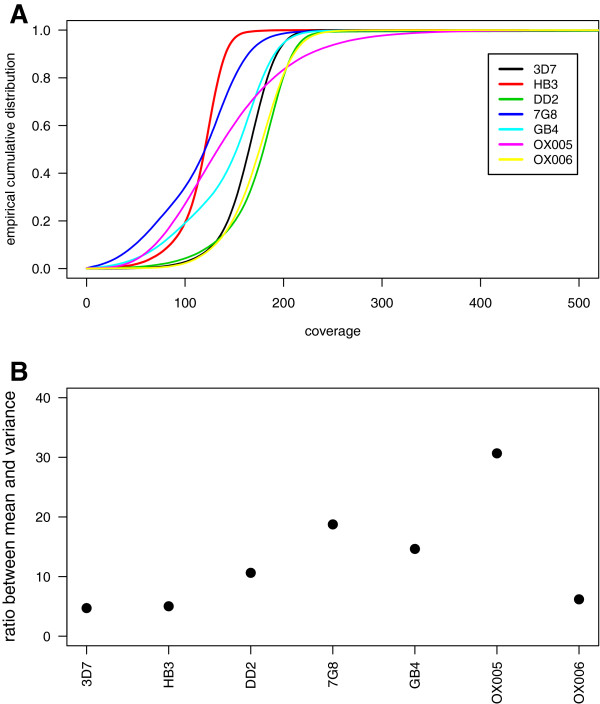
**Empirical coverage distributions are intrinsically overdispersed and skewed. A**. Observed coverage distributions. **B**. Overdispersion defined as the ratio between coverage mean and variance (see also Table 1).

Data under analysis comprises *Pf* samples of 5 well-characterised laboratory strains from different parts of the world (3D7 - reference strain of African origin, HB3 - Honduras, DD2 - Indonesia, 7G8 - Brazil, and GB4 - Ghana) and of 2 clinical isolates from travellers to Africa (OX005 - Ghana, and OX006 - Kenya). Each sample consists of millions of reads mapped onto the 3D7 reference genome (version 3.0, 23Mb, 19% GC content, Table 1), which was divided into non-overlapping 100-bp windows and filtered for the respective exome (120,309 100-bp windows in total). The resulting coverage distributions exhibit distinct shapes and summary statistics (Figure 1A and Table 1), thus the necessity of having flexible approaches for CNV detection. The percentage of windows with zero coverage or with coverage equal or greater than 500 reads is up to 0.51% (DD2), suggesting the presence of few CNVs in the samples.

**Table 1:** **Statistical description of the coverage distributions using 100-bp windows and after filtering the data for the *Pf * exome**

						**Coverage**			
**Samples**	**Origin**	**Number of reads**	**Mean**	**Variance**	**Range**	**=0**	**≤10**	**≥250**	**≥500**
3D7	Africa	19,590,258	162.8	767.4	0–794	1	3	25	14
HB3	Honduras	14,024,161	116.6	585.6	0–449	188	262	23	0
DD2	Indonesia	21,080,366	175.2	1861.9	0–749	139	214	873	470
7G8	Brazil	13,736,522	114.2	2141.2	0–794	188	1419	365	29
GB4	Ghana	17,157,171	142.6	2087.3	0–955	151	540	274	7
OX005	Ghana	17,214,916	143.1	4387.9	0–1386	187	308	7691	109
OX006	Kenya	20,850,309	173.3	1072.1	0–733	46	102	656	7

### Coverage distributions are intrinsically overdispersed, skewed, and long-tailed

A brief description of the empirical coverage distributions led to two key observations. First, every coverage distribution is characterised by extreme overdispersion as the variance is greater than the mean in each sample (Table 1 and Figure 1B). This statistical property seems intrinsic to coverage data because of its observation in the 3D7 resequencing data, where just a few hits should be identified under the assumption of no errors in sequencing, mapping, and in the annotated genome. Second, these distributions are skewed and long tailed as suggested by the skewness and kurtosis coefficients (Additional file 1).

### Coverage distributions are described well by a Poisson-Gamma model

To analyse the data, we devised a CNV detection strategy based on Poisson-Gamma and Poisson-Lognormal, two probability distributions known for their flexibility in tackling overdispersion. To estimate these models, we divided each coverage profile according to the respective GC content and analysed the corresponding data separately. The Poisson, Poisson-Lognormal and Poisson-Gamma distributions were then compared against each other being the latter the best model for the data irrespective of the criteria used (Additional file 2). According to this model, the expected coverage distributions are almost indistinguishable from the observed ones (Additional file 3). Therefore, the Poisson-Gamma distribution was used to determine robust CNV detection thresholds (shown in Additional file 4).

### The Poisson-Gamma approach shows a low baseline false positive rate

The baseline false positive rate of our method was first assessed through the analysis of the 3D7 resequencing data, where CNVs are known (*e.g.*, GTP cyclohydrolase I gene, PFL1155w), and few hits should be produced. The corresponding data analysis led to a proportion of hits lower than the statistical stringency used in the analysis (*e.g.*, 28 hits out of 120,309 100-bp windows using *γ*=99.9*%*; see Table 2). Specifically, we obtained 11 windows with too-low coverage, being sparsely distributed across the genome. Conversely we found 17 windows with too-high coverage, 13 of which are true positive hits located in the GTP cyclohydrolase I locus (Additional file 5), an amplified region already highlighted by CGH array technology [11]. When applied the FREEC software to the same data, there was a slightly lower proportion of hits (15/120,309) but this proportion can be increased by using alternative parameter settings. The cross-sample cn.MOPS approach was applied to all isolates (3D7, HB3, DD2, 7G8, GB4, OX005, and OX006), leading to the highest proportion of hits in 3D7 (358/120,309). Unlike our approach and FREEC, cn.MOPS could only partially detect the amplification of the GTP cyclohydrolase I gene locus (11 hits out of 13 possible windows).

**Table 2:** Analysis of real and simulated 3D7 resequencing data

	**Overall hits**					**PFL1155w locus**			
**Method**	**10**×^**∗**^	**20**×^**∗**^	**50**×^**∗**^	**Real data**		**10**×^**∗**^	**20**×^**∗**^	**50**×^**∗**^	**Real data**
PG with *γ*=99*%*	0.82%	0.58%	0.36%	0.08%		13	13	13	13
PG with *γ*=99.9*%*	0.19%	0.11%	0.05%	0.02%		13	13	13	13
FREEC	0.00%	0.00%	0.00%	0.01%		0	0	0	13
cn.MOPS ^+^	0.55%	0.16%	0.01%	0.30%		0	0	0	11

Results refers to the (mean) percentage of overall hits detected in relation to the total number of 100-bp windows (120,309) using real and simulated data from the 3D7 resequencing sample and the corresponding (average) number of 100-bp hits detected on the GTP cyclohydrolase I gene locus (PFL1155w, 1.3 kb in size).

^*^Results based on 10 independent simulated data sets.

^+^Analysis performed across samples (simulated or real where appropriate).

We extended the above analysis by studying the influence of read depth on the false positive rate in a simulation study. Ten independent samples were generated for each of 3 read depths (10×, 20×, and 50×). The results showed that the false positive rate of our method is lower or in line with the corresponding statistical stringency adopted in the analysis (Table 2). For example, using *γ*=99.9*%*, the proportion of hits was 0.19%, 0.11%, and 0.05% for 10×, 20×, and 50× read depths, respectively. Moreover, our method was able to detect the amplified GTP cyclohydrolase I gene locus in every generated data set. Conversely the FREEC software produced no CNV calls in each simulated data set, even when parameterised to target a higher number of CNVs. The FREEC software seems then less sensitive when using low read depth data and there are just a few hits to be detected. We applied cn.MOPS approach to the set of simulated samples with the same read depth and found a lower mean proportion of hits in comparison to our method running at *γ*=99*%*. However, when we increased stringency of our method (*γ*=99.9*%*), cn.MOPS was outcompeted. Finally, likewise FREEC, cn.MOPS could not identify the amplified GTP cyclohydrolase I gene locus in any of the generated samples.

### The Poisson-Gamma modelling approach detects known and novel CNV regions

**Figure 2:**
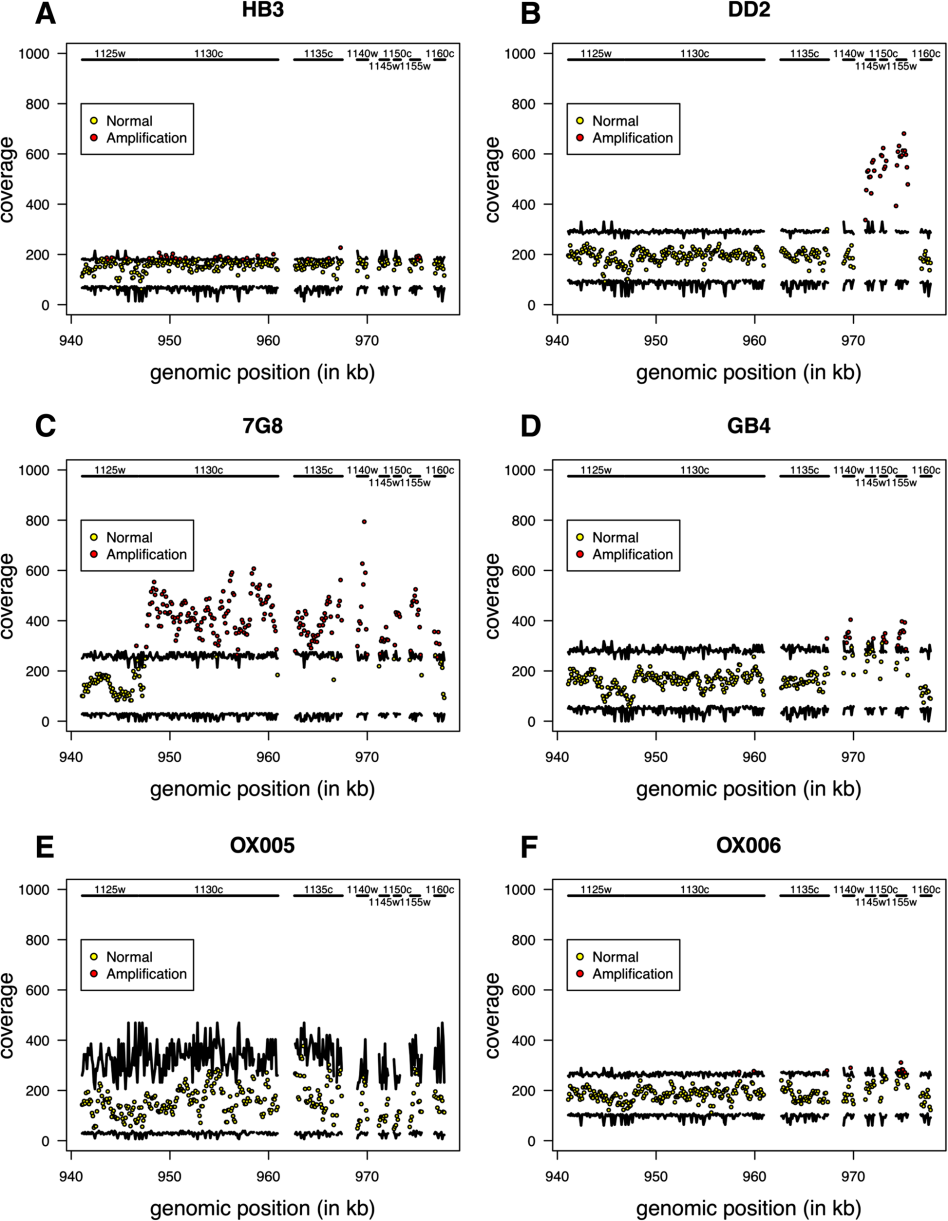
**Copy number variation between the PFL1125w and PFL1160w genes across different laboratory and clinical samples. A**. HB3 (Honduras); **B**. DD2 (Indonesia); **C**. 7G8 (Brazil); **D**. GB4 (Ghana); **E**. OX005 (Ghana); **F**. OX006 (Kenya). Note that the prefix PFL was removed from the corresponding gene names as available at genedb database (http://www.genedb.org).

The analysis of the remaining laboratory and clinical samples led to a total number of hits ranging from 257 (OX006) to 899 (DD2) using *γ*=99.9*%* (Table 3). The Poisson-Gamma approach could detect a large amplification located between the PFL1125w and PFL1160w genes (Figure 2A–D), which has been previously identified using CGH technology [11-13]. Another important CNV hit is the amplified region spanning the PFE1095w and PFE1160w genes in the Indonesian DD2 sample. This locus includes the PfMDR1 gene (PFE1150w) whose increased copy number is usually associated with multi-drug resistance of Southeast Asian *Pf* parasites [14-16]. Finally, a large deletion of the CLAG3.2 gene (PFC0110w) was also targeted in both Ghanaian samples (GB4 and OX005), a result in agreement with field reports from that country [17,18]. Other large hits can be found in Additional file 6.

**Table 3:** Summary of CNVs detected by the Poisson-Gamma model across different laboratory strains and clinical samples

		***γ=99%***				***γ=99.9%***			
**Sample**	**Type of CNV**	***#*Hits**	***#*Loci**	# **Gene**		***#*Hits**	***#*Loci**	**#Gene**	**Largest CNV(kb)**
HB3	Deletion	322	109	60		305	101	56	PFI1475 (2.0)
	Amplification	246	206	119		60	53	46	PF11_0503 (0.6)
DD2	Deletion	279	98	58		265	95	55	PFL2550w (1.7)
	Amplification	678	84	63		634	59	43	PFE1120w (14.8)
7G8	Deletion	243	125	83		205	101	61	MAL7P1.64 (1.1)
	Amplification	343	118	106		215	49	37	PFL1130w (6.7)
GB4	Deletion	262	98	48		253	92	45	PFC0110w (2.5)
	Amplification	108	84	79		47	38	36	PFL1155w (0.6)
OX005	Deletion	308	87	49		274	73	39	PFC0110w (2.8)
	Amplification	1019	772	516		192	140	118	PFD0669c (1.0)
OX006	Deletion	170	65	35		167	62	33	PF07_0013 (1.3)
	Amplification	277	226	188		90	70	64	MAL8P1.42 (1.1)

Results refer to the number of individual hits (i.e., 100-bp windows) and loci (pooled hits where contiguous) using the credible levels *γ* = 99% and 99.9% in the analysis.

### Comparison with FREEC and cn.MOPS approaches

The FREEC and cn.MOPS approaches were applied to the same laboratory and clinical samples; see Additional file 6 for a list of hits identified by these alternative methods. Table 4 shows the number of shared and exclusive hits of FREEC and cn.MOPS in relation to our approach (see also Additional files 7 and 8). For FREEC, the proportion of shared hits varies with the sample under analysis, ranging from 27.4% (GB4) to 63.9% (DD2) for deletions and from 4.2% (OX005) to 84.3% (DD2) for amplifications (using *γ*= 99.9% in our method). The high proportion of shared amplifications in the Indonesian DD2 sample is mainly due to the strong signal derived from the PfMDR1 locus. Our method produced a higher number of exclusive hits, most of them in loci not targeted by FREEC, including a large amplification between PFL1125w and PFL1160c genes in HB3 lab strain previously validated by CGH technology [11]. Conversely the majority of the FREEC-exclusive hits, except those from the OX005 sample, are located in genomic regions targeted by our methodology, filling ’gaps’ or increasing the size of the putative CNVs (Additional file 7). The low frequency of FREEC-exclusive hits in these ’shared’ loci shows that little is missed by not including a formal merging algorithm in our method. In fact, the procedure of simply merging adjacent hits was enough to reduce significantly the number of hits produced by our method (Table 3).

**Table 4:** Hits shared and exclusively detected by the Poisson-Gamma (PG) model, the FREEC and the cn.MOPS approaches

		**PG with *γ*=99.9% vs FREEC**				**PG with *γ*=99.9% vs cn.MOPS**		
**CNV**	**Sample**	$S_{\mathrm{PG}-\text{FREEC}}$	$E_{\mathrm{PG}}$	$E_{\mathrm{FREEC}}$		$S_{\mathrm{PG}-\mathrm{cn.MOPS}}$	$E_{\mathrm{PG}}$	$E_{\mathrm{cn.MOPS}}$
Deletions	HB3	175 (55.2)	130 (41.0)	12 (3.8)		195 (63.5)	110 (35.8)	2 (0.7)
	DD2	175 (63.9)	90 (32.8)	9 (3.3)		152 (57.4)	113 (42.6)	0 (0.0)
	7G8	81 (29.1)	124 (44.6)	73 (26.3)		120 (21.4)	85 (15.1)	357 (63.5)
	GB4	72 (27.4)	181 (68.8)	10 (3.8)		150 (50.0)	103 (34.3)	47 (15.7)
	OX005	153 (51.0)	121 (40.3)	26 (8.7)		205 (69.3)	69 (23.3)	22 (7.4)
	OX006	93 (55.0)	74 (43.8)	2 (1.2)		62 (37.1)	105 (62.9)	0 (0.0)
Amplifications	HB3	19 (29.7)	41 (64.1)	4 (6.3)		23 (12.2)	37 (19.7)	128 (68.1)
	DD2	586 (84.3)	48 (6.9)	61 (8.8)		608 (85.2)	26 (3.6)	80 (11.2)
	7G8	187 (37.6)	28 (5.6)	283 (56.8)		212 (17.8)	3 (0.3)	973 (81.9)
	GB4	6 (10.5)	41 (71.9)	10 (17.5)		38 (11.8)	9 (2.8)	274 (85.4)
	OX005	62 (4.2)	130 (8.8)	1291 (87.1)		168 (5.7)	24 (0.8)	2473 (93.5)
	OX006	27 (22.9)	63 (53.4)	28 (23.7)		64 (20.9)	26 (8.5)	216 (70.6)

The frequencies (and the respective percentages in brackets) refer to the number of hits shared and exclusively detected by the PG model against FREEC and cn.MOPS, whereS_*PG-FREEC*_ andS_*PG-cn.MOPS*_ denote the hits shared between the respective pair of methods,E_*PG*_**,**E_*FREEC*_ andE_*cn.MOPS*_ denote the exclusive hits produced by the corresponding methodology in the respective comparison. Percentages are in relation to the overall number of deletions and amplifications identified by the respective pair of methods.

In the case of cn.MOPS, the proportion of shared hits ranges from 21.4% (7G8) to 69.3% (OX005) for deletions and from 5.7% (OX005) to 85.2% (DD2) for amplifications (using *γ*=99.9*%* in our method). Likewise in the above comparison, the high proportion of shared amplifications in DD2 laboratory strain also results from hits in the PfMDR1 locus. With respect to exclusive hits, our method tends to produce a higher number of these than cn.MOPS for deletions, but a lower number for amplifications. This observation suggests that our method tends to define higher thresholds than cn.MOPS for deletions, but lower thresholds for amplifications. However, it is difficult to generalise this result due to differences in stringency definition employed by each method.

Finally, the FREEC software running under default settings could not detect a large amplification between PFL1125w and PFL1160c genes in HB3 isolate identified by our method (Figure 2A) and validated by CGH technology [11]. Similarly, the cn.MOPS approach could not detect the amplification of the GTP cyclohydrolase I gene in the 3D7 re-sequencing data. However, we could recover some of these false negatives, and possible others, using alternative (non-default) parameter settings (results not shown). This result demonstrates the possibility of missing important CNVs by applying these approaches, specifically, when one needs to set them up ’blindly’ in the case of new isolates, large sample sizes, or when their underlying assumptions are not fully met by the data.

### Validation of coverage-based hits using CGH array data

The validity of coverage-based hits produced by each methodology was assessed using published CGH data (Table 5). For HB3 and DD2 lab strains, where we used a previously compiled list of CGH hits [19], the FREEC software led to the highest concordance (92.9%) between coverage- and CGH-based hits. This apparent best performance of FREEC is mainly due to a lesser number of hits produced by this software. The use of greater stringency in our method (*i.e.*, *γ*=99.9*%*) led to an increase in the corresponding concordance, from 75.9% to 78.9% in HB3, and from 89% to 92.6% in DD2. For 7G8 and GB4 strains, where we re-analysed the original CGH data, our methodology outperformed FREEC and cn.MOPS overall and, in the best case scenario (*γ*=99.9*%* in our method), we could confirm 78.3% (7G8) and 54.0% (GB4) of our hits. The cn.MOPS approach seems inferior to our method and FREEC, with the lowest concordance rates irrespective of the lab strain and CNV type. This result may be explained by the nature of the data under analysis (*i.e.*, far from being Poisson distributed when there is no CNV present) and the small sample size.

**Table 5:** Hits shared between CGH and coverage data using the Poisson-Gamma (PG) model, the FREEC software, and the cn.MOPS approach

**Strain**	**Methodology**	**Deletions**	**Amplifications**	**Overall**
HB3	FREEC	—	—	195/210 (92.9%)
	cn.MOPS	—	—	214/348 (61.5%)
	PG with *γ*=99*%*	—	—	431/568 (75.9%)
	PG with *γ*=99.9*%*	—	—	288/365 (78.9%)
DD2	FREEC	—	—	792/831 (95.3%)
	cn.MOPS	—	—	746/840 (88.8%)
	PG with *γ*=99*%*	—	—	854/957 (89.0%)
	PG with *γ*=99.9*%*	—	—	826/899 (91.9%)
7G8	FREEC	89/154 (57.8%)	285/470 (60.6%)	374/624 (59.9%)
	cn.MOPS	91/477 (19.1%)	236/1185 (19.9%)	327/1662 (19.7%)
	PG with *γ*=99*%*	164/243 (67.5%)	216/343 (63.0%)	380/586 (64.9%)
	PG with *γ*=99.9*%*	153/205 (75.6%)	176/215 (81.9%)	329/420 (78.3%)
GB4	FREEC	32/82 (39.0%)	4/16 (25.0%)	36/98 (36.7%)
	cn.MOPS	77/197 (39.1%)	28/273 (10.3%)	105/470 (22.3%)
	PG with *γ*=99*%*	152/262 (59.0%)	24/108 (22.2%)	176/370 (47.6%)
	PG with *γ*=99.9*%*	148/253 (58.5%)	14/47 (29.8%)	162/300 (54.0%)

CGH hits of HB3 and DD2 lab strains were taken from Samarakoon *et al.*[19], while CGH hits of 7G8 and GB4 lab strains were obtained by re-analysing the corresponding original data available from Jiang *et al.*[37]. The percentages in brackets are in relation to the total number of coverage hits produced by the corresponding method.

## Discussion

We have proposed a Poisson hierarchical modelling approach for CNV detection, which is flexible and robust to the common problem of overdispersed coverage data. Using simulation and resequencing data of the 3D7 reference genome, we have demonstrated a low baseline false positive rate of the methodology across different read depth. However, this low baseline false positive rate needs to be assessed in other genomic settings, preferably where reference resequencing data is available, or potentially using a robust simulation strategy with realistic statistical assumptions and parameter settings. In general, one can reduce the baseline false positive rate of any coverage-based method if mapping distance information is also taken into account. True positive hits are then likely to be those whose coverage and mapping distance analyses agree with each other. In particular, strong evidence for deletions is provided from genomic regions with too-low coverage and average mapping distance greater than expected, while amplified regions entail extremely high coverage and average mapping distance less than expected [3]. This integrated data analysis, whilst increasing robustness and accuracy, remains to be developed.

The proposed approach was also applied to non-reference strain data and identified a large number of CNVs that could be validated by CGH data. The empirical and simulation results have demonstrated that our approach may be applicable to larger genomes where read depths can be lower, or in settings where overdispersion is present [4,10]. Recent deep sequencing technologies are currently generating data with high coverage (>50-fold on average) irrespective of the organism under study. Thus, we do not expect that the accuracy and robustness of our methodology shown here would change in these cutting-edge data sets with similar genomic coverage. In practice, the potentially high computational burden associated with larger genomes or strong overdispersion is surmountable by implementing parallel computing techniques.

Our method seemed to outperform FREEC and cn.MOPS approaches with respect to concordance of hits confirmed by CGH data for 7G8 and GB4 strains. However, a more accurate comparison was compromised by difficulties in relating stringency. The stringency of our method is controlled by the credibility level, a rigorous statistical parameter, but more difficult to be inferred in algorithms that do not consider a specific statistical model, as in the FREEC software. Notwithstanding this difficulty, we showed that increasing the stringency of our methodology led to a high concordance with the FREEC-based hits. However, the FREEC software could only detect a known amplification at the GTP cyclohydrolase locus in HB3 [11] using alternative parameter setting. The dependency of findings on parameter setting is mitigated in our methodology as the analyst only needs to specify the underlying stringency.

The Poisson hierarchical modelling approach has the advantage of handling with different data patterns but, as it stands, cannot estimate the corresponding copy number. To overcome this limitation, one can invoke a proportionality between mean (or median) coverage and the underlying copy number, as assumed elsewhere [4-6]. In theory, this assumption requires modelling the coverage distributions through a finite mixture of Poisson-Gamma (or Poisson-Lognormal) distributions where each component is associated with a given copy number, as implemented in the cn.MOPS approach but using a Poisson mixture model. A Monte Carlo Markov Chain method with reversible jumps [20] can be applied to the corresponding Bayesian estimation. Whilst the greater computational overhead involved in applying this more complex model is surmountable, its utility would rely on having sufficient data to estimate the additional parameters. In our setting, the very low number of hits detected suggests that data might be too scarce to fit models with increased complexity. Our approach seems then the perfect balance between model complexity and data information, thus a potentially useful addition to bioinformatic toolkits used to identify CNVs from sequence coverage data.

## Conclusions

We have developed a robust Poisson hierarchical modelling approach for CNV detection using sequence coverage data. When applied to the *Pf* genome, the method shows a low false positive rate in the 3D7 resequencing data, and is able to detect known and putative novel CNV regions. This promising result suggests the application of this approach to different settings, such as the human or other micro-organism genomes. Because the approach was developed under a strong but flexible statistical framework, it brings increased statistical rigour and robustness into the problem of CNV detection. In addition, it will allow important extensions, such as the estimation of the underlying copy number.

## Methods

### Sequence data and processing

Data consists of 7 *Pf* genomes of which 5 are of well-characterised laboratory strains (3D7 - reference strain of African origin, HB3 - Honduras, DD2 - Indonesia, 7G8 - Brazil, and GB4 - Ghana) and 2 are of clinical origin (OX005 - Ghana, OX006 - Kenya) [21]. The 3D7 reference strain data was used to assess the baseline false positive rate. All seven genomes underwent whole genome sequencing on the Illumina Genome Analyzer II (54/76-base paired read) platform and processed as described elsewhere [21]. In brief, multiple alignment (bam) files were generated from fastq files of each whole genome sequencing data set after mapping the reads onto the 3D7 reference genome (version 3.0) using the *smalt* software (http://www.sanger.ac.uk/resources/software/smalt/). Using the toolkit SAMtools, poorly mapped reads were removed from the analysis. The corresponding raw data sets are publicly available from the European Bioinformatics Institute website (http://www.ebi.ac.uk, SRA study ERP0000190) and the PlasmoDB database (http://www.plasmodb.org).

### Estimating coverage profiles

In each sample, calculation of coverage profile followed the usual procedure for human data [4-6]. The 3D7 reference genome was first partitioned into non-overlapping and equal-size windows. The size of each window was set at 100 bp as it seemed a good compromise between sufficient resolution for CNV detection and reasonable statistical properties of the data. In each window, we calculated the respective coverage as the number of mapped reads using their starting mapped positions. Since coverage can be confounded by the GC content, we also calculated the underlying GC content profile using the FREEC software. Windows within 100kb of subtelomeric and centromeric regions, as well as windows with poor mapping score, were excluded from the analysis, since they could introduce bias due to putative mapping errors [22]. Windows associated with antigenic diversity gene families (including vars, stevors, and rifins) were also discarded owing to the fact that they are intrinsically variable [11]. We also filtered out non-coding regions, thus focusing the analysis on the *Pf* exome as on average there is twice as much coverage in coding regions [21]. After this filtering process, the coverage profile of each target genome comprises 120,309 100-bp windows accounting for nearly 53% of the 3D7 reference genome. We adjusted our results for GC content by splitting each coverage profile into separate data sets comprising the coverage values of windows with similar GC content and analysing them separately [4,5]. Since the GC content distribution does not follow a Uniform distribution in the 3D7 reference genome [23], we divided the coverage profile according to the 5%-centiles (5%, 10%, 15%,…) of the GC content distribution as a way to ensure similar statistical power across the different set of windows.

### Detection of CNVs using a Poisson hierarchical modelling approach

When analysing each data set, we specified the following Multinomial distribution for the coverage values of windows with similar GC content

(1) $$f\left(\left\{n_{g,i}\right\}|N_{g};\eta \right)=N_{g}!\underset{i}{\prod }\frac{p_{g}{\left(i\right)}^{n_{g,i}}}{n_{g,i}!},$$

where$N_{g}=\sum_{i}n_{g,i}$is the total number of windows with GC content *g*, *n*_*g*,*i*_ is the total number of windows with coverage *i* and GC content *g*, *p*_*g*_(*i*) is the overall probability of mapping *i* reads onto any window with GC content *g*. The probabilities *p*_*g*_(*i*) are usually described by a Poisson distribution as long as the reads are independently and equally distributed across the genome [4]. Because of overdispersion, we extended this model by varying its mean parameter according to another probability distribution. We chose the Gamma or Lognormal distributions to model the variation of the Poisson mean parameter due to their flexibility and successful application across different scientific fields [24-27]. These two second-level distributions, when conjugated with the Poisson model, give rise to the so-called Poisson-Gamma (with parameters *α* and *β*) and Poisson-Lognormal (with parameters *μ* and *σ*^2^), respectively. Mathematically, the Poisson-Gamma model is given by

(2) $$p_{g}(i)=\frac{\Gamma (\alpha +i)}{\Gamma (i)\Gamma (\alpha +1)}{\left(\frac{\beta }{\beta +1}\right)}^{\alpha }{\left(\frac{1}{\beta +1}\right)}^{i},$$

where *α* and *β* are the shape and rate parameters of a Gamma distribution, respectively. The Poisson-Lognormal distribution does not show closed-form expression but good numerical algorithms exist for its calculation when applied to a specific data set [28].

The estimation of these two models was performed through Bayesian methods using non-informative prior distributions for the respective parameters. With respect to the Poisson-Gamma, we used a Gamma prior distribution for the parameters *α* and *β*. The respective shape and scale parameters were set at 0.001, as often specified in Bayesian applications (see, for example, WinBUGS online documentation at http://www.mrc-bsu.cam.ac.uk/bugs). For the Poisson-Lognormal model, we set a Gaussian prior distribution with mean 0 and standard deviation 10^4^ for the parameter *μ*, and a Gamma prior distribution with the shape and scale parameters equal to 0.001 for the parameter 1/*σ*^2^, respectively. To obtain posterior samples through parallel computing, we used WinBUGS (http://www.mrc-bsu.cam.ac.uk/bugs) and JAGS (http://www.mcmc-jags.sourceforge.net/) coupled with the R software through the R2WinBUGS and R2JAGS packages [[29]. The respective R and WinBUGS/JAGS scripts are available from the authors upon request.

After obtaining the posterior parameter samples, the models were tested against each other using Bayes factors and the Deviance Information Criteria (DIC) [30]. With respect to Bayes factors, we calculated the underlying prior predictive probability (PPPs) of each model using the so-called BIC-MC estimator, which seems to provide robust and stable results under hierarchical modelling [31]. To this end, we generated posterior samples for the log-likelihood function required for the calculation of BIC-MC estimator. We performed this task in WinBUGS/JAGS for the Poisson-Gamma distribution since the corresponding likelihood function is known analytically. However, for the case of the Poisson-Lognormal model whose probability distribution has no closed-form expression, we calculated the posterior samples of the log-likelihood function using subroutines available in the package PAM for the R software [27].

For the formal CNV detection, we used the corresponding posterior predictive distribution, which embodies all uncertainty regarding coverage given the observed data and prior information. The calculation was performed through the simulation of ’new’ coverage values according to the respective posterior parameter samples and the best model for the data. We then determined the corresponding HPD credible interval at *γ*=99% and 99.9% using Chen-Shao method [32] in order to set appropriate CNV detection limits. In particular, windows with coverage greater than the upper bound of HPD credible interval are likely to contain amplifications. Conversely windows with coverage lesser than the lower bound of HPD credible interval are deemed deletions. To reduce the false positive rate associated with deletions, we removed all putative hits whose coverage was greater than zero in every nucleotide position. Data of total coverage per position was obtained using the SAMtools (samtools.sourceforge.net). The final step of the analysis comprised merging contiguous hits. In theory, we could have introduced a more formal merging stage by adapting, for example, the popular Circular Binary Segmentation algorithm [33] for sequence coverage data. In practice, we did not intend ’forcing’ our method to generate larger genomic regions artificially. A recent study shows that simply merging contiguous hits is sufficient to generate a small number of loci in relation to the total number of hits [34]. Another reason for not including such formal procedure is due to the fact that its application is in rigour compromised while studying the exome (*i.e.*, a ’fragmented’ version of a genome) of a given organism. In this case, formal merging can only be performed at the gene level.

### Detection of CNVs using FREEC and cn.MOPS softwares

There are several CNV detection methods currently available in the literature [6]. We chose to compare our methodology against FREEC and the cn.MOPS, two potentially promising approaches when applied to human genome data [6]. However, the performance of the cross-sample cn.MOPS approach is likely to be impaired because: (1) even that this approach invokes a finite mixture of Poisson distributions for the read counts of a given segment, it reverts to a Poisson distribution when there is no CNV present, a distribution that does not fully agree with our data; (2) since the corresponding analysis is performed across samples, the accuracy of the method is dependent on the sample size. In this regard, Klambauer et al. [6] suggested a minimum number of 6 samples for the cn.MOPS to work well. This minimum sample size seems rather low in comparison to those typically found in the literature of this type of modelling approach [35,36].

In general, the FREEC software divides the reference genome into non-overlapping and equal-size windows, and calculates the corresponding coverage profile of the target sample. A polynomial regression model is then used to describe the dependency between coverage and GC content. The respective predicted values are first standardised and then smoothed out. The final stage of the analysis consists of estimating the copy number in each segment and merging the regions with similar copy number. With this purpose, the software assumes that the ploidy of the organism under analysis is known and the copy number of a given segment is proportional to the median coverage of all the windows with similar GC content. For the *Pf* sequence coverage data, we specified ploidy of 1 and used a quadratic regression model. We also analysed the data through a cubic regression model, as often suggested for human genome data [5], but obtained unrealistic copy number distributions (results not shown). The parameters of the segmentation algorithm were set at their default setting.

The cn.MOPS approach is also based on sequence coverage data partitioned into non-overlapping windows. It assumes a finite mixture of Poisson distributions (with a known number of components) for the coverage across samples of any given window. In this approach, each component of the mixture describes the coverage distribution associated with a given copy number under the assumption of a linear relationship between mean coverage and copy number. The model is fitted to each segment via an EM algorithm and the most probable component determined. To apply this approach to our data, we set the copy number to be an integer from 0 to 4, where the value 1 is the ’normal’ copy number (or the ploidy of the organism under study). The remaining parameters were specified at their default settings as explained in the documentation of the respective R package (called cn.MOPS).

### Simulation study based on 3D7 resequencing data

To assess the baseline false positive rate of our method, we performed a simulation study based on 3D7 resequencing data. We generated 10 independent data sets from the 3D7 resequencing sample according to read depths of 10 ×, 20×, and 50 ×, corresponding to a total of 1.25, 2.5, and 6 million reads, respectively. Each data set refers to the coverage profile of 120,309 100-bp windows and was simulated according to a Multinomial distribution with a sample size given by the corresponding total number of reads associated with a specific read depth and probability vector defined by the relative coverage profile of the original 3D7 reference sample. We analysed each data set separately using our method and the FREEC software. In the former, we used the Poisson-Gamma (PG) distribution and two different credible levels (*γ*=99*%* and 99.9*%*), while in the latter we analysed the data using the default parameter setting; we tested alternative parameter sets but the corresponding results were qualitatively similar (not shown). To summarise the results, we calculated the mean percentage of detected hits in relation to the total number of windows under analysis. Finally, we analysed each batch of 10 independent samples of a given read depth altogether using the cn.MOPS software and compared the results to those obtained from the analysis of each individual sample.

### Comparative genomic hybridisation array data

To assess the reliability of the coverage-based hits, we brought into the analysis available CGH data for the HB3, DD2, 7G8 and GB4 laboratory strains. In the first two strains, we used a pre-compiled list of CGH hits [19], identifying the corresponding 100-bp windows used in the coverage analysis. In the last two strains, we re-analysed the original CGH data [37] accessible from NCBI’s Gene Expression Omnibus through GEO Series accession number [GEO:GSE25656]. Data refers to log2-ratio between intensities of these two strains in relation to those obtained from the 3D7 reference strain. We used the software SnoopCGH as a visualisation tool [38]. After removing probes in regions not considered in our coverage data analysis, we applied the following strategy to detect CNVs: (1) obtain a segmented profile of each individual intensity data set using DNAcopy package for the R software, (2) divide each segmented intensity profile into non-overlapping windows of size 100-bp as done in the coverage data analysis, (3) calculate the corresponding empirical distribution of segmented intensities, and (4) compute the respective highest probability density interval with a given probability mass *τ* (say *τ*=95%), and (5) produce a CNV call whenever the intensity of a given window is not included in this interval. In this regard, a negative log2-ratio intensity was considered to be indicative of a deletion while a positive log2-ratio was deemed a putative amplification. For each pairwise comparison, concordance rates were calculated for each data set and defined as the number of CNV hits identified by coverage analysis and confirmed by the CGH technology divided by the total number of coverage-based hits.

## Competing interests

The authors declare that they have no competing interests.

## Authors’ contributions

AP, SGC and TGC conceived the project. NS developed the Poisson hierarchical modelling approach and wrote the first draft of the manuscript, and modifications were performed by AP, CJS, SGC and TGC. NS, SAA and TGC performed the data analysis. CJS and SGC obtained and processed the clinical samples. All authors have read and approved the final version of the manuscript.

## Supplementary Material

Additional file 1 Skewness and kurtosis of empirical coverage distributions. Click here for file

Additional file 2 **Statistical model comparison between Poisson, Poisson-Gamma, and Poisson-Lognormal distributions.** The Poisson and Poisson-Lognormal models were compared to the Poisson-Gamma using the Deviance Information Criteria (DIC) [30] and Bayes factors (BF). In the case of DIC, we calculated the ratio between that of the Poisson-Gamma and those of the remaining models. With respect to BF, they were estimated as the log-ratio between the corresponding predictive prior probabilities via the BIC-MC estimator [31]. Click here for file

Additional file 3 **Expected and empirical cumulative coverage distributions.** Expected coverage distributions refer to the corresponding posterior predictive distributions for the set of all 100-bp windows used in the analysis. Click here for file

Additional file 4 **Limits for CNV detection used on each sample as function of the underlying GC content.** CNV detection limits were determined according to the posterior predictive probability distribution of the Poisson-Gamma (the best model for every data set under analysis). Click here for file

Additional file 5 A large amplification detected between PFL1125w and PFL1160w genes in the 3D7 reference genome data using the Poisson-Gamma model. Click here for file

Additional file 6 **CNVs larger than 500 bp detected using the Poisson-Gamma model (*γ=99%*), the FREEC software, and cn.MOPS approach.** Click here for file

Additional file 7 Comparison between hits detected by the Poisson-Gamma model and the FREEC software. Click here for file

Additional file 8 Ternary diagrams plotting the joint proportions of shared and exclusively detected hits by the PG model, the FREEC software, and cn.MOPS. Click here for file
