# Supplementary figures and images for: A Poisson hierarchical modelling approach to detecting copy number variation in sequence coverage data
Source: BMC Genomics. 2013 Feb 26;14:128. doi: 10.1186/1471-2164-14-128 (PMC3679970; doi:10.1186/1471-2164-14-128)

# Additional file 1

## Skewness

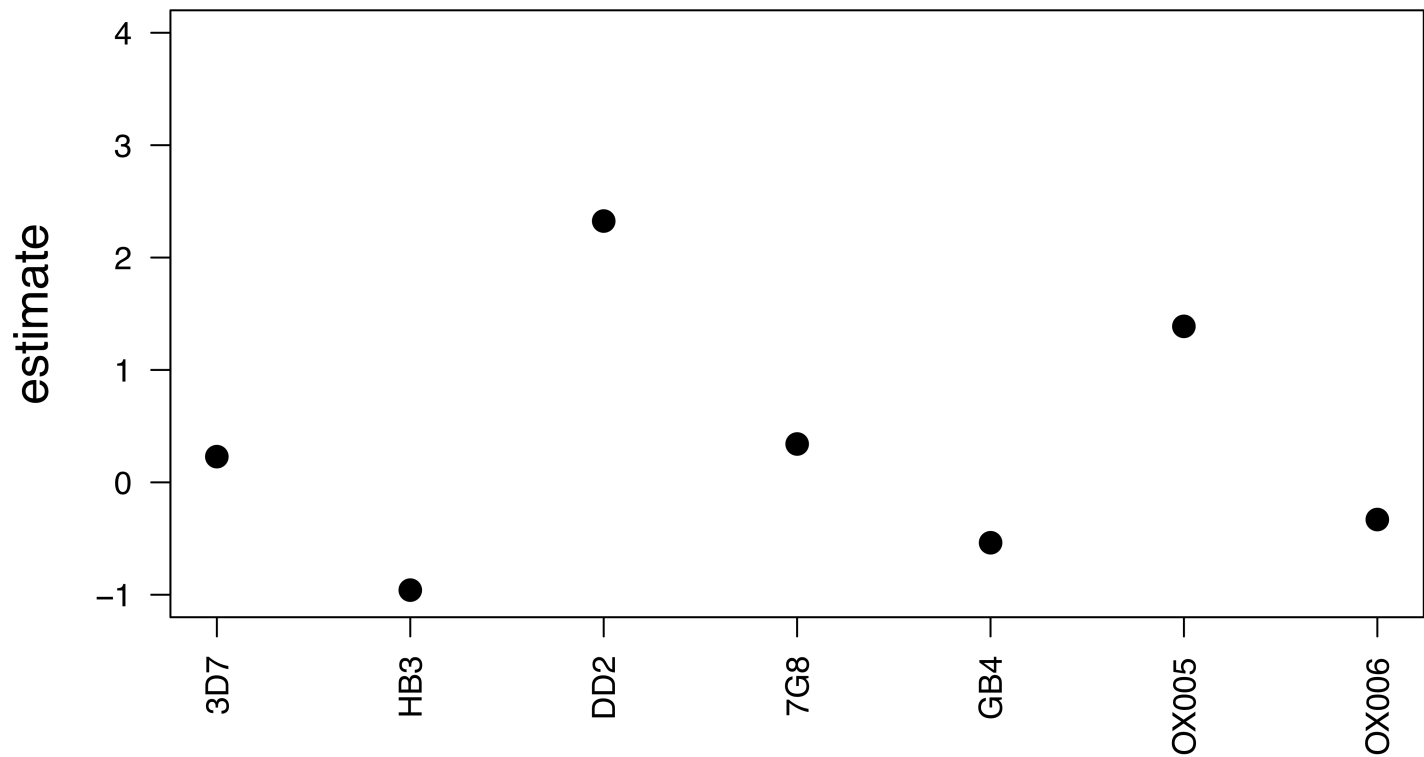

## Kurtosis

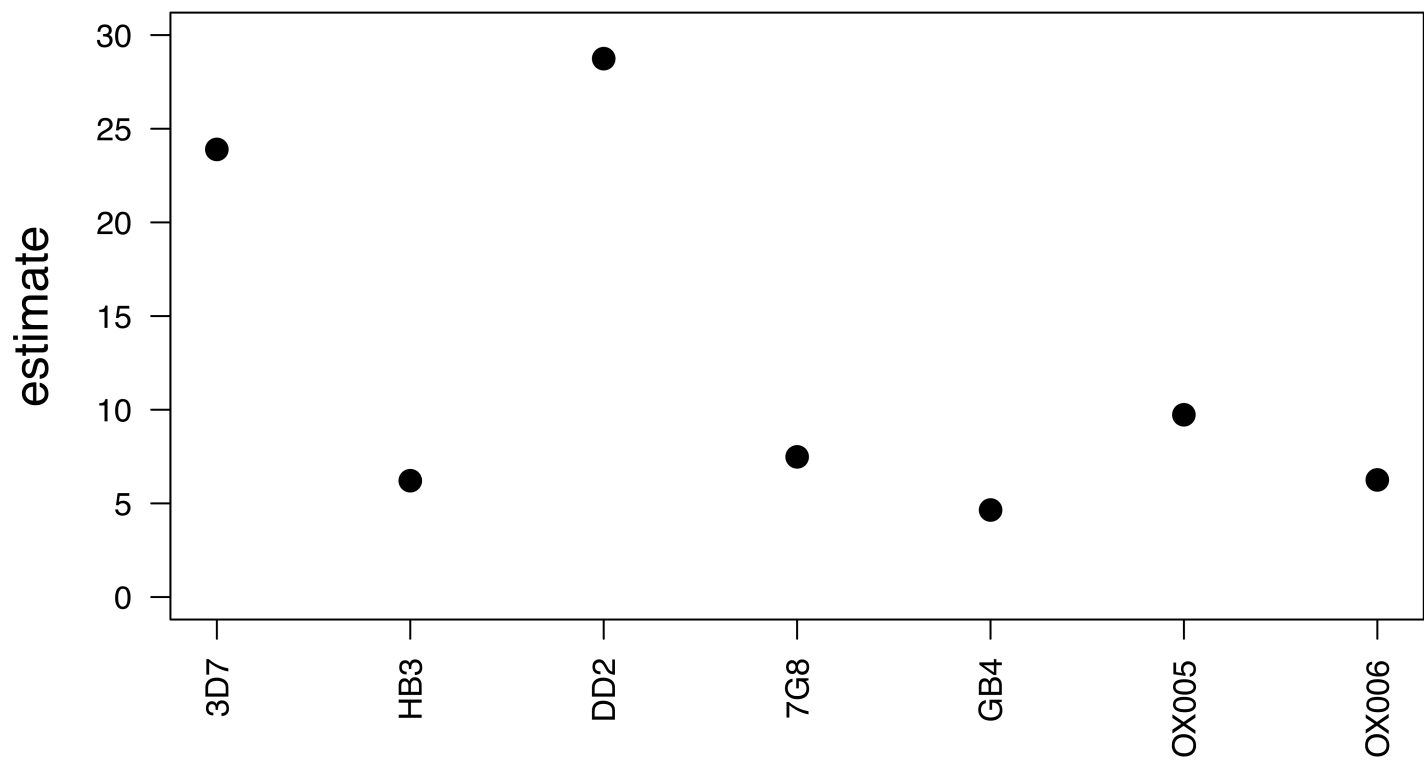

Supplement: Additional file 1 — Skewness and kurtosis of empirical coverage distributions. [file 1471-2164-14-128-S1.pdf]

# Additional file 3

**3D7**

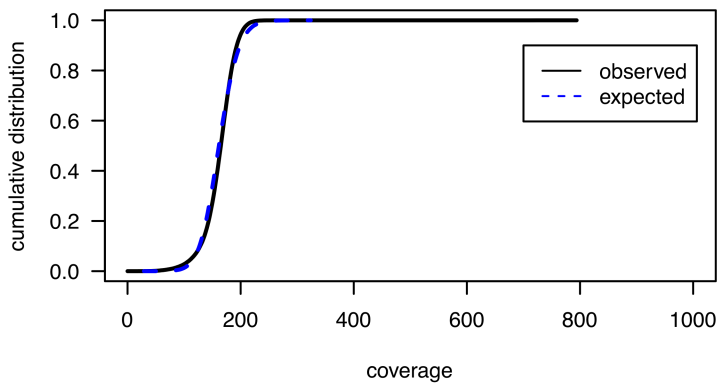

**HB3**

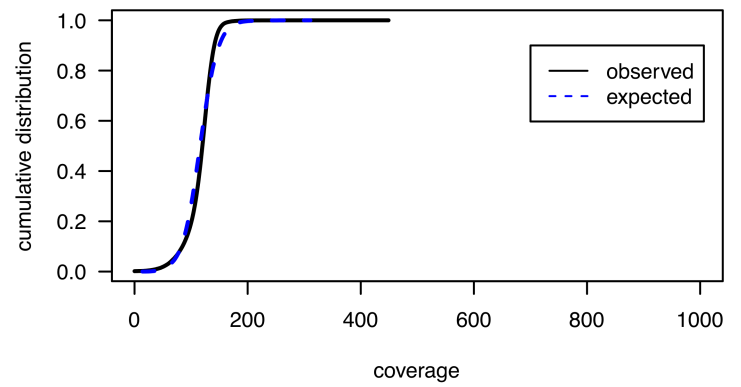

**DD2**

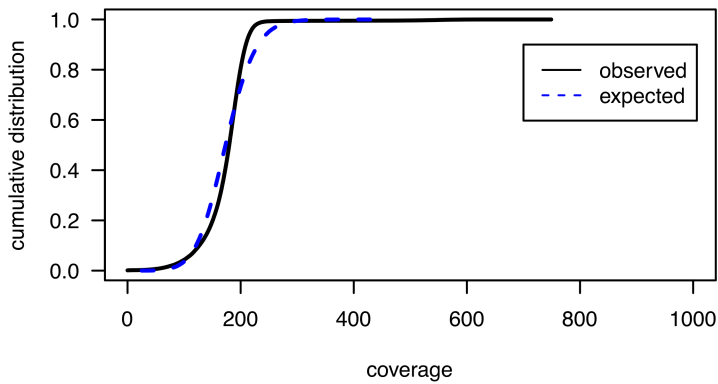

**7G8**

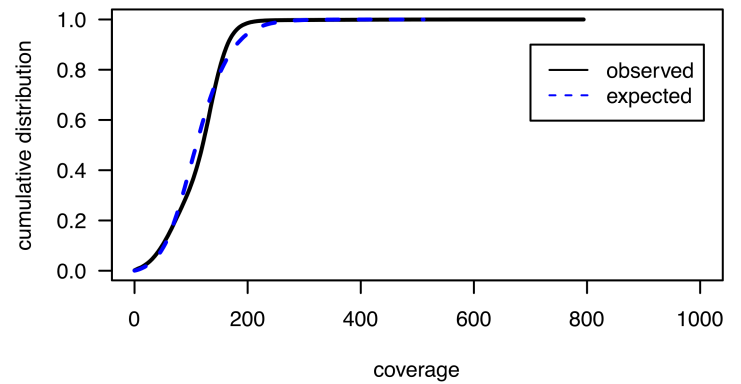

**GB4**

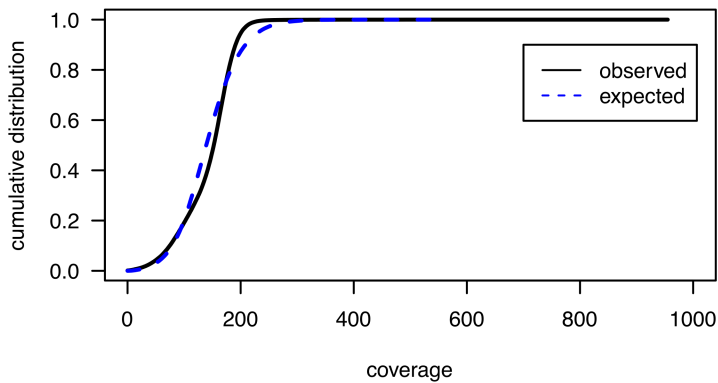

**OX005**

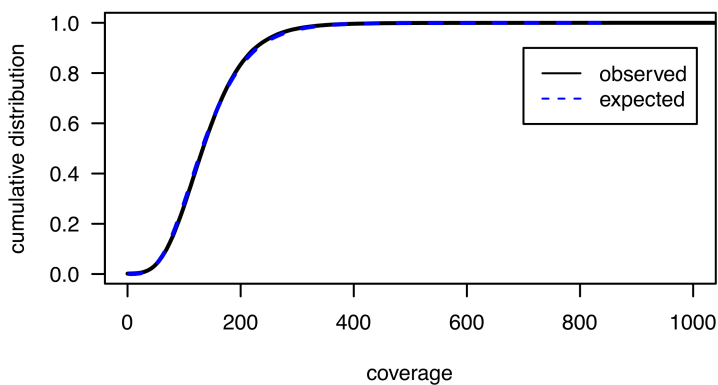

**OX006**

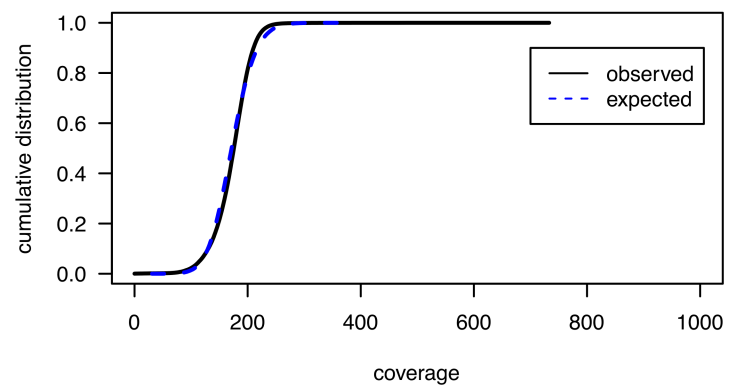

Supplement: Additional file 3 — Expected and empirical cumulative coverage distributions. Expected coverage distributions refer to the corresponding posterior predictive distributions for the set of all 100-bp windows used in the analysis. [file 1471-2164-14-128-S3.pdf]

# Additional file 4

**3D7**

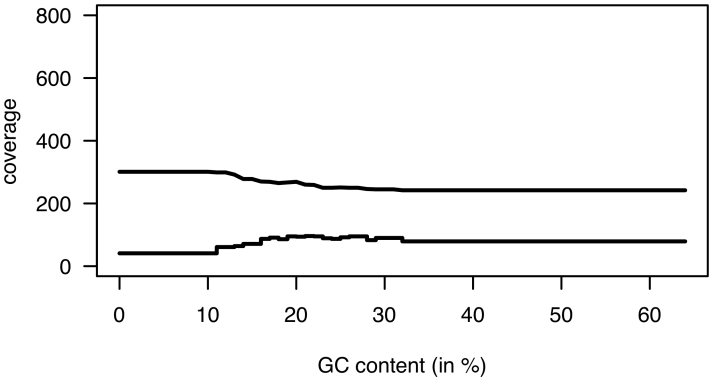

**HB3**

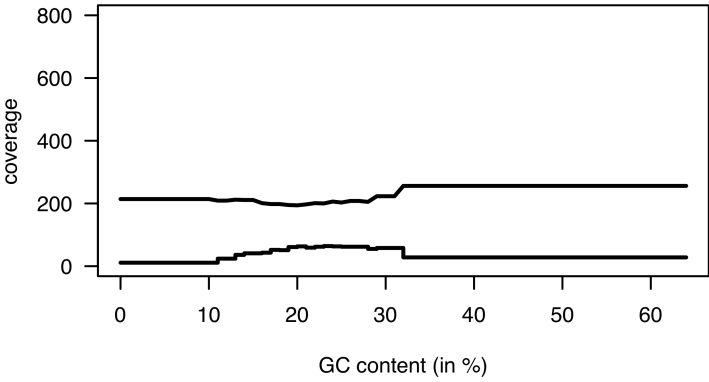

**DD2**

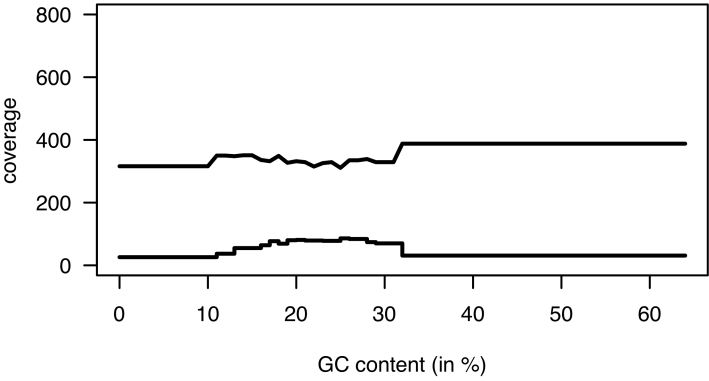

**7G8**

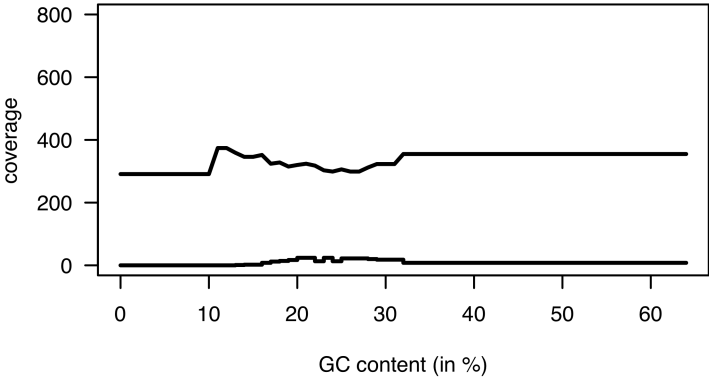

**GB4**

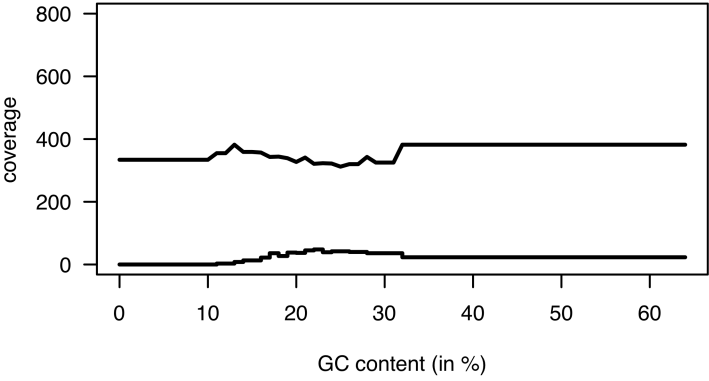

**OX005**

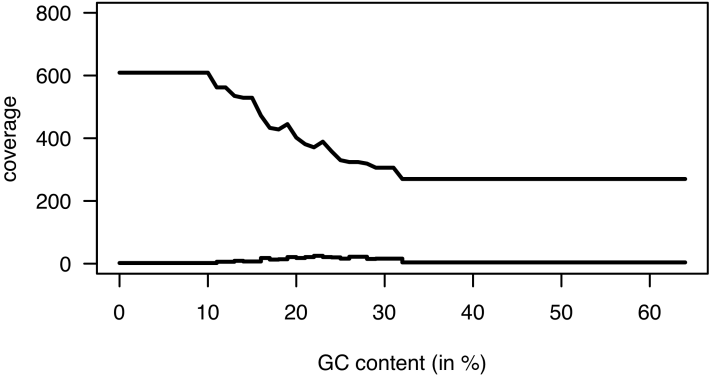

**OX006**

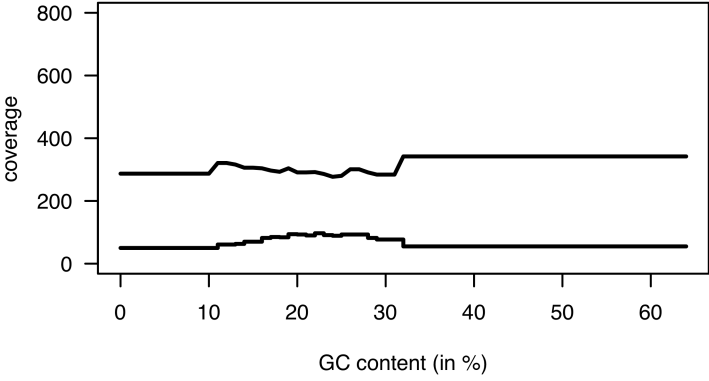

Supplement: Additional file 4 — Limits for CNV detection used on each sample as function of the underlying GC content. CNV detection limits were determined according to the posterior predictive probability distribution of the Poisson-Gamma (the best model for every data set under analysis). [file 1471-2164-14-128-S4.pdf]

# Additional file 5

**3D7**

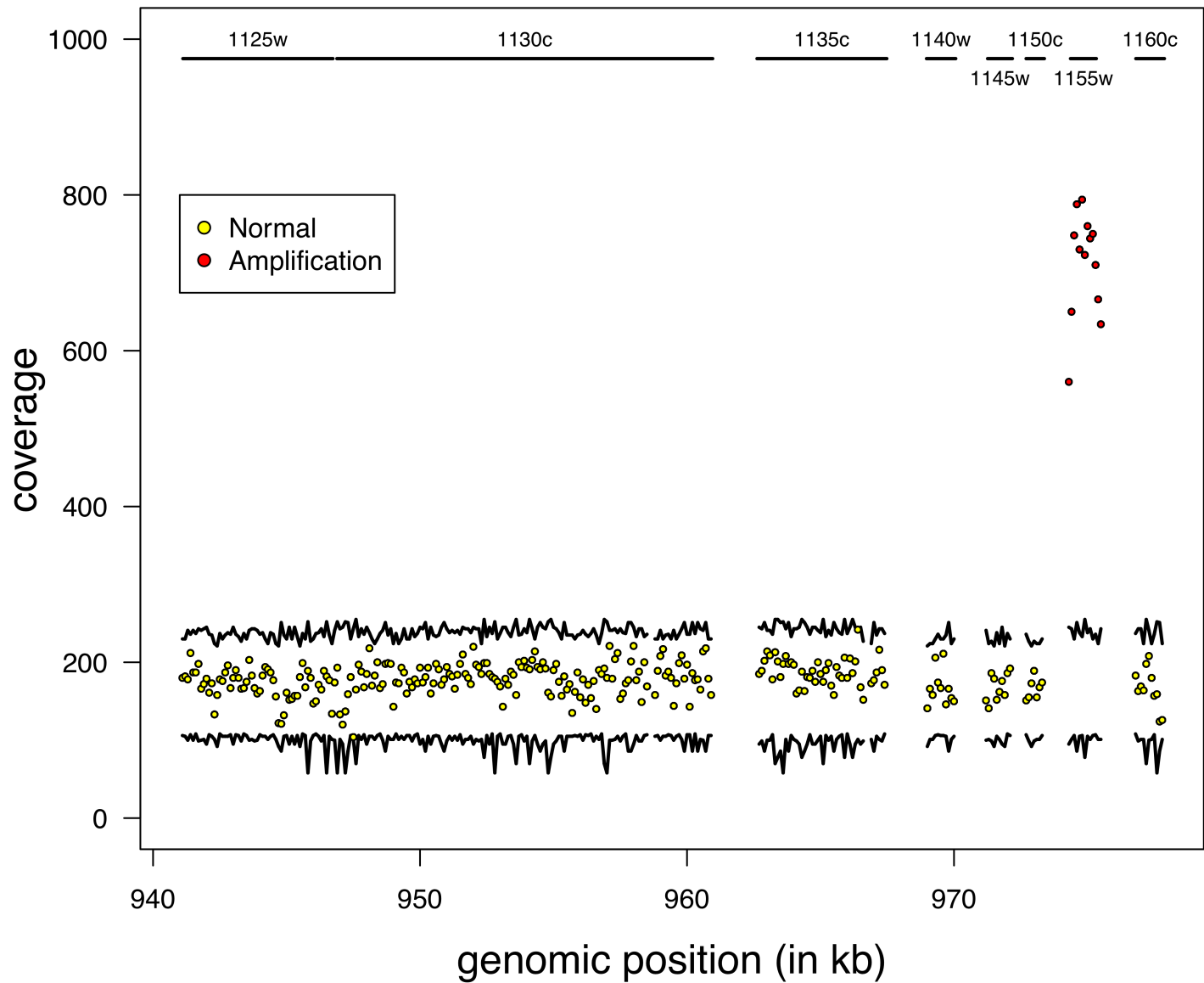

Supplement: Additional file 5 — A large amplification detected between PFL1125w and PFL1160w genes in the 3D7 reference genome data using the Poisson-Gamma model. [file 1471-2164-14-128-S5.pdf]
