# Supplementary material for: A Poisson hierarchical modelling approach to detecting copy number variation in sequence coverage data
Source: BMC Genomics. 2013 Feb 26;14:128. doi: 10.1186/1471-2164-14-128 (PMC3679970; doi:10.1186/1471-2164-14-128)
Supplement: Additional file 2 — Statistical model comparison between Poisson, Poisson-Gamma, and Poisson-Lognormal distributions. The Poisson and Poisson-Lognormal models were compared to the Poisson-Gamma using the Deviance Information Criteria (DIC) [30] and Bayes factors (BF). In the case of DIC, we calculated the ratio between that of the Poisson-Gamma and those of the remaining models. With respect to BF, they were estimated as the log-ratio between the corresponding predictive prior probabilities via the BIC-MC estimator [31]. [file 1471-2164-14-128-S2.pdf]

Additional file 2

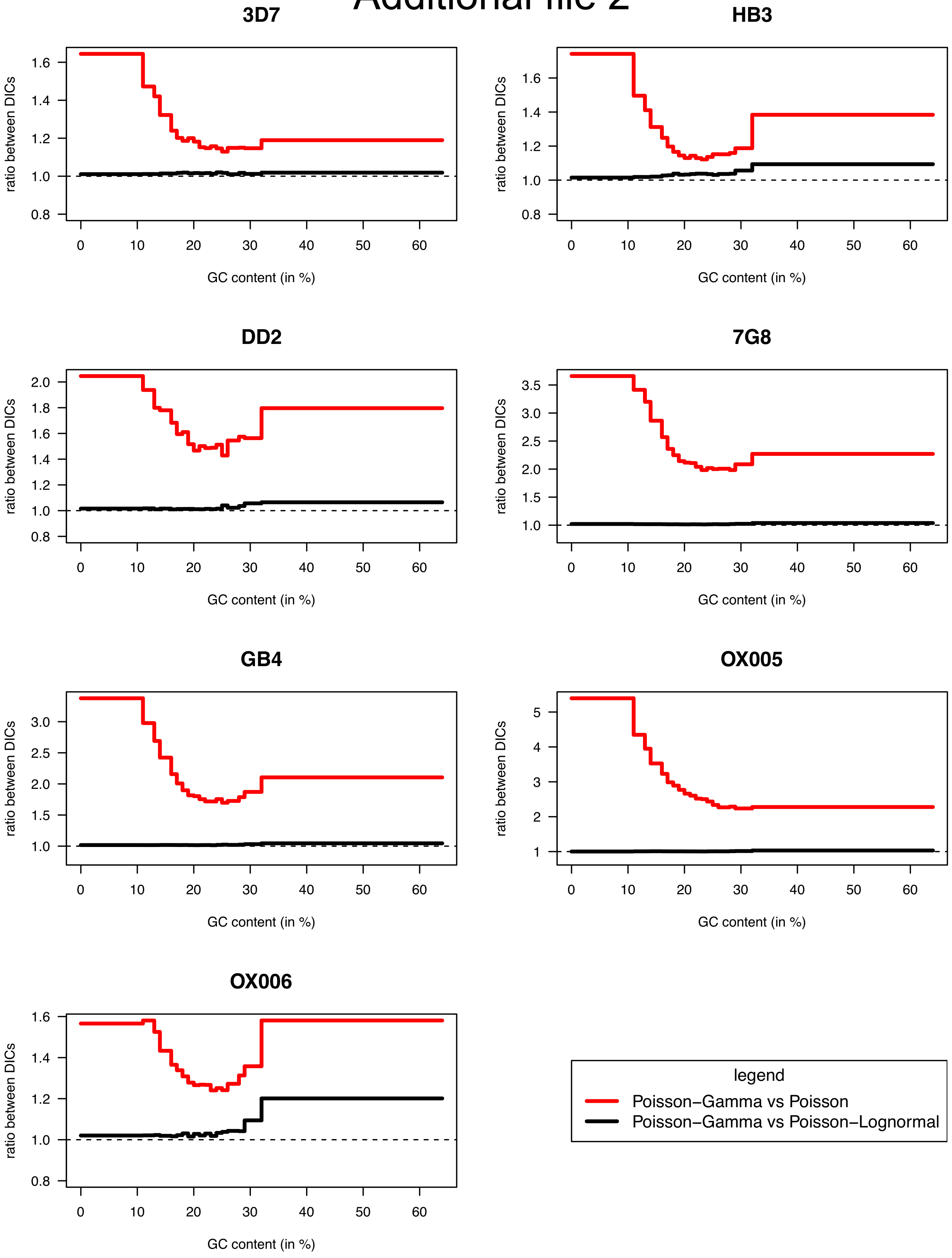

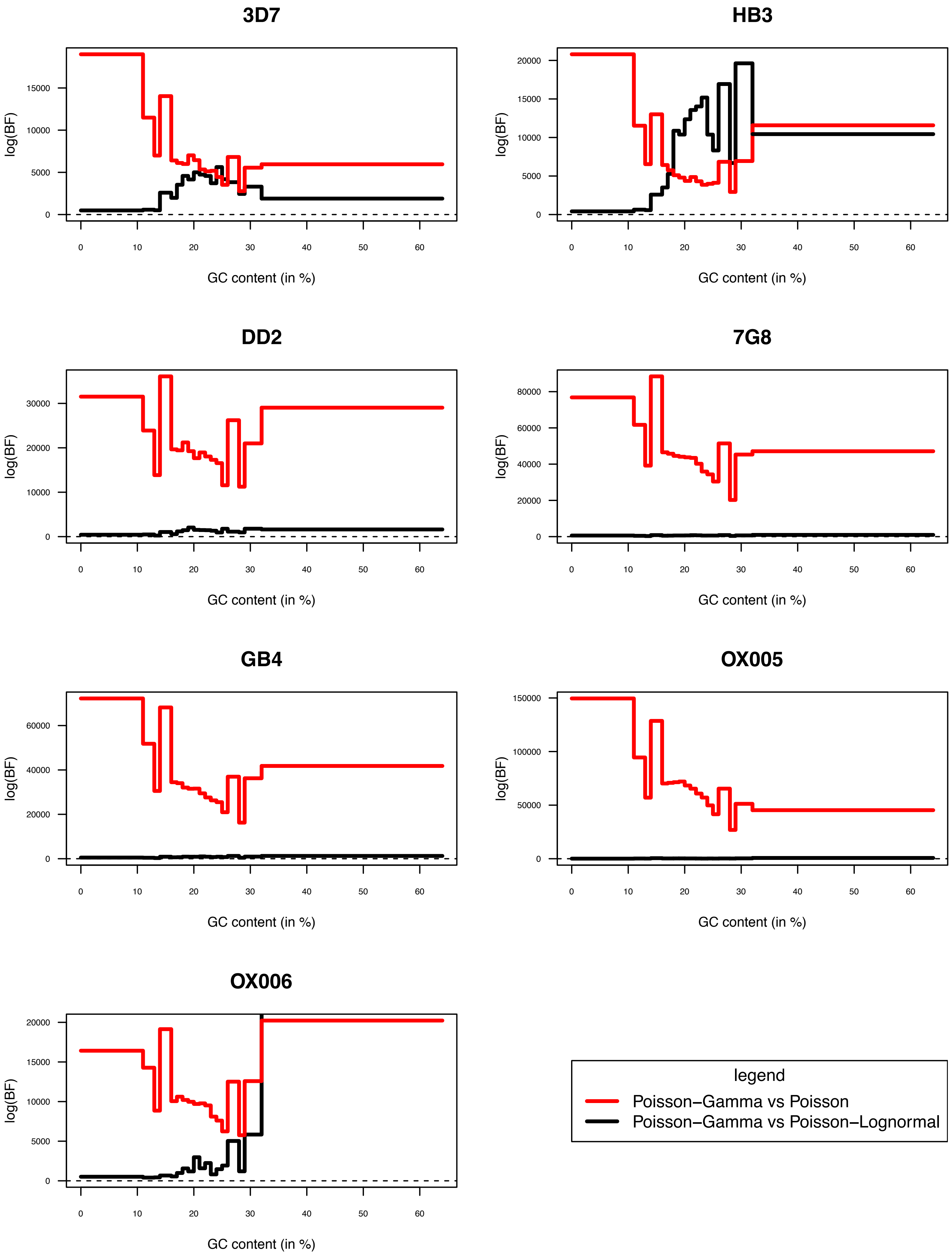
3D7
HB3
DD2
7G8
GB4
OX005
OX006
log(BF)
GC content (in %)
0
10
20
30
40
50
60
15000
10000
5000
20000
30000
80000
60000
40000
150000
100000
50000
legend
Poisson–Gamma vs Poisson
Poisson–Gamma vs Poisson–Lognormal
