# Supplementary material for: A Poisson hierarchical modelling approach to detecting copy number variation in sequence coverage data
Source: BMC Genomics. 2013 Feb 26;14:128. doi: 10.1186/1471-2164-14-128 (PMC3679970; doi:10.1186/1471-2164-14-128)
Supplement: Additional file 6 — CNVs larger than 500 bp detected using the Poisson-Gamma model (γ=99%), the FREEC software, and cn.MOPS approach. [file 1471-2164-14-128-S6.pdf]

Additional file 6
CNVs (larger than 500 bp) detected using the Poisson-Gamma approach

HB3	Chromosome	Begin	End	Gene name	Coverage	Size (in bp)
Deletions	4	559901	560600	PFD0620c	9	700
	4	615301	616300	PFD0660w	0	1000
	4	616901	618200	PFD0665c	4	1300
	7	129401	130000	PF07_0013	14	600
	7	130801	131600	PF07_0013	29	800
	7	612401	613500	MAL7P1.64	11	1100
	8	430401	431000	MAL8P1.36	0	600
	8	434601	435800	PF08_0033	35	1200
	8	436301	437500	MAL8P1.38	12	1200
	8	443301	444100	PF08_0034	48	800
	8	462601	463200	PF08_0035	12	600
	9	1202901	1204900	PFI1475w	48	2000
	9	1205401	1206600	PFI1475w	34	1200
	10	1413601	1414100	PF10_0348	5	500
	14	3139301	3140900	PF14_0732	0	1600
	14	3142001	3144000	PF14_0733	0	2000
Amplifications	11	1934701	1935300	PF11_0503	1668	600

DD2	Chromosome	Begin	End	Gene name	Coverage	Size (in bp)
Deletions	1	515101	515800	PFA0650w	71	700
	1	532501	533100	PFA0665w	22	600
	2	104201	105200	PFB0100c	0	1000
	7	101601	102200	PF07_0007	0	600
	7	129401	130200	PF07_0013	40	800
	7	131101	131900	PF07_0013	60	800
	7	612401	613500	MAL7P1.64	51	1100
	7	651301	651800	PF07_0056	10	500
	8	434401	435800	PF08_0033	44	1400
	8	436301	437100	MAL8P1.38	18	800
	8	443401	444100	PF08_0034	27	700
	8	461401	461900	PF08_0035	52	500
	8	462101	463100	PF08_0035	53	1000
	12	2168101	2169800	PFL2550w	0	1700
Amplifications	5	888601	891700	PFE1095w	16394	3100
	5	892101	894800	PFE1095w	13710	2700
	5	896001	896500	PFE1100w	2152	500
	5	897501	899800	PFE1105c	12163	2300
	5	901401	902200	PFE1110w	3722	800
	5	903001	903700	PFE1115c	3368	700
	5	907801	922600	PFE1120w	80312	14800
	5	922801	928100	PFE1120w	28664	5300
	5	928501	936400	PFE1120w	42366	7900
	5	940201	941700	PFE1130w	7964	1500
	5	942701	943300	PFE1135w	2836	600
	5	944401	945000	PFE1140c	2839	600
	5	948901	953600	PFE1145w	25634	4700
	5	957801	962000	PFE1150w	22595	4200
	5	963201	964500	PFE1155c	6738	1300
	5	966101	969300	PFE1160w	17627	3200
	12	971301	972200	PFL1145w	4654	900
	12	972701	973400	PFL1150c	3984	700
	12	974301	975600	PFL1155w	7491	1300

7G8	Chromosome	Begin	End	Gene name	Coverage	Size (in bp)
Deletions	1	532501	533200	PFA0665w	17	700
	4	138901	139400	PFD0110w	1	500
	4	559901	560700	PFD0620c	14	800
	7	612401	613500	MAL7P1.64	0	1100
	7	651301	651800	PF07_0056	6	500
	8	434401	435500	PF08_0033	12	1100
	8	436501	437500	MAL8P1.38	7	1000
	8	443401	444100	PF08_0034	6	700
	8	461401	461900	PF08_0035	10	500
	8	462101	463100	PF08_0035	35	1000
	10	1413601	1414100	PF10_0348	4	500
Amplifications	12	947701	954400	PFL1130c	28811	6700
	12	955101	956800	PFL1130c	7870	1700
	12	957001	957800	PFL1130c	2948	800
	12	957901	958600	PFL1130c	3415	700
	12	958801	960800	PFL1130c	9315	2000
	12	962801	963300	PFL1135c	2012	500
	12	963401	964100	PFL1135c	2714	700
	12	965001	966500	PFL1135c	6176	1500
	12	967001	967500	PFL1135c	2321	500
	12	969101	970000	PFL1140w	4527	900
	12	972901	973400	PFL1150c	2131	500
	12	974501	975400	PFL1155w	4156	900

GB4	Chromosome	Begin	End	Gene name	Coverage	Size (in bp)
Deletions	1	514201	514700	PFA0650w	0	500
	1	515101	515800	PFA0650w	25	700
	1	532601	533100	PFA0665w	27	500
	3	121401	121900	PFC0110w	10	500
	3	122301	124800	PFC0110w	4	2500
	4	616901	617500	PFD0665c	0	600
	7	131301	131900	PF07_0013	27	600
	7	612901	613500	MAL7P1.64	38	600
	7	627501	628100	PF07_0053	93	600
	7	628501	629100	PF07_0053	91	600
	8	430401	431000	MAL8P1.36	0	600
	8	434401	435200	PF08_0033	48	800
	8	435301	435800	PF08_0033	22	500
	8	436301	437500	MAL8P1.38	22	1200
	8	441401	441900	PF08_0034	19	500
	8	443401	444100	PF08_0034	2	700
	8	461401	461900	PF08_0035	11	500
	8	462101	463400	PF08_0035	6	1300
Amplifications	12	974701	975300	PFL1155w	2213	600

OX005	Chromosome	Begin	End	Gene name	Coverage	Size (in bp)
Deletions	1	514101	514700	PFA0650w	0	600
	1	515101	515700	PFA0650w	2	600
	3	121401	121900	PFC0110w	1	500
	3	122001	124800	PFC0110w	7	2800
	4	560001	560700	PFD0620c	3	700
	4	615301	616300	PFD0660w	0	1000
	4	616901	618200	PFD0665c	0	1300
	4	988101	989100	PFD1030c	1	1000
	4	989201	990500	PFD1030c	9	1300
	7	129401	130100	PF07_0013	36	700
	7	131301	131900	PF07_0013	17	600
	7	612701	613500	MAL7P1.64	16	800
	8	434401	435800	PF08_0033	0	1400
	8	436501	437500	MAL8P1.38	0	1000
	8	441501	442000	PF08_0034	1	500
	8	443001	444100	PF08_0034	8	1100
	8	461401	461900	PF08_0035	4	500
	8	462101	463400	PF08_0035	42	1300
Amplifications	4	649801	650800	PFD0669c	7961	1000
	8	301301	302200	MAL8P1.19	7486	900
	13	1510901	1511400	PF13_0210	3441	500

OX006	Chromosome	Begin	End	Gene name	Coverage	Size (in bp)
Deletions	7	129401	130200	PF07_0013	26	800
	7	130601	131900	PF07_0013	128	1300
	7	612401	613300	MAL7P1.64	82	900
	7	651301	651800	PF07_0056	117	500
	8	434701	435800	PF08_0033	120	1100
	8	436301	437500	MAL8P1.38	102	1200
	8	443401	444100	PF08_0034	45	700
	8	461401	461900	PF08_0035	27	500
	8	462301	463400	PF08_0035	54	1100
	13	1429401	1430000	MAL13P1.176	3	600
Amplifications	8	455901	457000	MAL8P1.42	4813	1100

CNVs larger than 500 bp detected according to the FREEC software using default settings.

HB3	Chromosome	Begin	End	Gene name	Coverage	Size (in bp)
Deletions	7	129401	130000	PF07_0013	14	600
	7	130801	131600	PF07_0013	29	800
	7	612401	613500	MAL7P1.64	11	1100
	8	462601	463400	PF08_0035	76	800
	9	1202901	1204900	PFI1475w	48	2000
	9	1205401	1206600	PFI1475w	34	1200
	14	3139301	3140900	PF14_0732	0	1600
	14	3142001	3144000	PF14_0733	0	2000
Amplification	11	1934701	1935300	PF11_0503	1668	600

DD2	Chromosome	Begin	End	Gene name	Coverage	Size (in bp)
Deletions	2	104201	105200	PFB0100c	0	1000
	7	101601	102200	PF07_0007	0	600
	7	612401	613500	MAL7P1.64	51	1100
	7	651301	651800	PF07_0056	10	500
	8	434401	435800	PF08_0033	44	1400
	8	436301	437500	MAL8P1.38	50	1200
	8	443401	444100	PF08_0034	27	700
	8	461401	461900	PF08_0035	52	500
	8	462101	463200	PF08_0035	89	1100
	12	2168101	2169800	PFL2550w	0	1700
Amplifications	5	888601	891700	PFE1095w	16394	3100
	5	892101	894800	PFE1095w	13710	2700
	5	896001	897000	PFE1100w	3434	1000
	5	897401	899800	PFE1105c	12490	2400
	5	901401	902200	PFE1110w	3722	800
	5	902601	904900	PFE1115c	9237	2300
	5	907801	922600	PFE1120w	80312	14800
	5	922801	928300	PFE1120w	29164	5500
	5	928401	936400	PFE1120w	42695	8000
	5	937201	938100	PFE1125w	3569	900
	5	940201	941700	PFE1130w	7964	1500
	5	942701	943300	PFE1135w	2836	600
	5	944401	945000	PFE1140c	2839	600
	5	948901	953700	PFE1145w	25913	4800
	5	957801	962200	PFE1150w	22936	4400
	5	963201	964500	PFE1155c	6738	1300
	5	966101	969300	PFE1160w	17627	3200
	7	618401	619000	MAL7P1.66	2038	600
	8	453501	454800	MAL8P1.41	3628	1300
	8	455401	457100	MAL8P1.42	4463	1700

7G8	Chromosome	Begin	End	Gene name	Coverage	Size (in bp)
Deletions	7	612401	613500	MAL7P1.64	0	1100
	8	434401	435800	PF08_0033	39	1400
	8	436301	437500	MAL8P1.38	24	1200
	8	441401	441900	PF08_0034	70	500
	8	443001	444100	PF08_0034	146	1100
	8	461401	461900	PF08_0035	10	500
	8	462101	463400	PF08_0035	74	1300
	13	2445801	2446500	PF13_0323	8	700
Amplifications	2	104201	105200	PFB0100c	1281	1000
	7	618401	619000	MAL7P1.66	953	600
	7	622201	622700	MAL7P1.67	1262	500
	9	240701	242300	PFI0250c	2771	1600
	11	604601	605300	PF11_0168	1186	700
	12	932901	934200	PFL1115w	1677	1300
	12	934901	938700	PFL1120c	5546	3800
	12	941101	958600	PFL1125w	55979	17500
	12	958801	961000	PFL1130c	9785	2200
	12	962701	966700	PFL1135c	14811	4000
	12	966901	967500	PFL1135c	2567	600
	12	969001	970100	PFL1140w	5091	1100
	12	971201	972200	PFL1145w	3116	1000
	12	972701	973400	PFL1150c	2682	700
	12	974301	975600	PFL1155w	5127	1300
	12	976801	977700	PFL1160c	2525	900
	12	1260001	1261500	PFL1480w	2747	1500
	14	1185401	1186000	PF14_0280	1851	600
	14	2134801	2136000	PF14_0495	2545	1200

GB4	Chromosome	Begin	End	Gene name	Coverage	Size (in bp)
Deletions	3	121401	121900	PFC0110w	10	500
	3	122001	124800	PFC0110w	4	2800
	7	129401	130200	PF07_0013	49	800
	7	131201	131900	PF07_0013	55	700
	8	461401	461900	PF08_0035	11	500
	8	462101	463400	PF08_0035	6	1300
Amplifications	14	1185401	1186000	PF14_0280	1803	600

OX005	Chromosome	Begin	End	Gene name	Coverage	Size (in bp)
Deletions	4	559901	560700	PFD0620c	8	800
	4	615301	616300	PFD0660w	0	1000
	4	616901	618200	PFD0665c	0	1300
	4	987801	990500	PFD1030c	21	2700
	7	612601	613500	MAL7P1.64	65	900
	7	618401	618900	MAL7P1.66	47	500
	8	434401	435800	PF08_0033	0	1400
	8	436301	437500	MAL8P1.38	11	1200
	8	441501	442000	PF08_0034	1	500
	8	443001	444100	PF08_0034	8	1100
	8	455401	455900	MAL8P1.42	49	500
	8	456601	457100	MAL8P1.42	167	500
	8	461401	461900	PF08_0035	4	500
	8	462101	463400	PF08_0035	42	1300
Amplifications	4	232501	233300	PFD0205c	2475	800
	4	234001	237100	PFD0207c	6705	3100
	5	194801	195500	PFE0235c	2939	700
	5	505601	506700	PFE0570w	3543	1100
	5	525001	527300	PFE0590w	5758	2300
	5	665401	666100	PFE0800w	1947	700
	5	718701	719600	PFE0855c	2518	900
	5	779101	779700	PFE0935c	2413	600
	5	968501	969300	PFE1160w	2285	800
	6	327101	328200	PFF0380w	2818	1100
	6	344601	347200	PFF0410w	5834	2600
	6	365601	366700	PFF0440w	3721	1100
	6	492201	493000	PFF0575c	1441	800
	6	493201	495300	PFF0575c	4204	2100
	6	495501	499000	PFF0575c	8017	3500
	6	499201	500300	PFF0575c	2110	1100
	6	659501	660500	PFF0765c	3182	1000
	6	970501	971000	PFF1145c	2029	500
	7	362701	363200	MAL7P1.23	1853	500
	7	864301	864900	PF07_0081	3345	600
	7	866901	867600	PF07_0082	2784	700
	7	901901	902800	MAL7P1.111	2449	900
	7	904101	904700	PF07_0086	1673	600
	7	1053101	1054400	PF07_0104	3919	1300
	7	1152201	1153200	MAL7P1.149	2955	1000
	8	300001	300500	PF08_0018	2834	500
	8	301301	303500	MAL8P1.19	13154	2200
	8	647201	648000	MAL8P1.70	2511	800
	8	664601	665200	MAL8P1.73	2305	600
	8	685601	686700	PF08_0060	3679	1100
	8	860501	861400	PF08_0089	4523	900
	8	916301	916800	PF08_0094	1768	500
	8	982701	983400	MAL8P1.113	2185	700

(continued - OX005)

OX005	Chromosome	Begin	End	Gene name	Coverage	Size (in bp)
Amplifications	9	415401	416400	PFI0440w	3482	1000
	9	865201	866600	PFI1040c	3082	1400
	9	866901	870200	PFI1040c	7674	3300
	9	1049701	1051100	PFI1280c	2885	1400
	9	1051401	1052200	PFI1280c	1362	800
	9	1052601	1053800	PFI1280c	3333	1200
	9	1054601	1056000	PFI1280c	3324	1400
	9	1056501	1057700	PFI1280c	2839	1200
	9	1108801	1110800	PFI1335w	4846	2000
	10	399501	400200	PF10_0096	1446	700
	10	400301	401100	PF10_0096	2084	800
	10	445701	446600	PF10_0113	2329	900
	10	586001	588900	PF10_0143	6113	2900
	10	1156601	1157100	PF10_0273	1551	500
	10	1441901	1442600	PF10_0357	2138	700
	11	881601	882100	PF11_0240	2261	500
	11	1205101	1205700	PF11_0324	2314	600
	11	1256901	1257400	PF11_0333	1504	500
	11	1492201	1496200	PF11_0392	9307	4000
	12	234401	235700	PFL0270c	2978	1300
	12	242801	245800	PFL0275w	4841	3000
	12	246501	247300	PFL0275w	1903	800
	12	908501	909300	PFL1085w	2185	800
	12	909501	913300	PFL1085w	9681	3800
	12	1366801	1370100	PFL1600c	8769	3300
	12	1886101	1887600	PFL2165w	3249	1500
	12	1888201	1889500	PFL2170c	3080	1300
	12	1889801	1892400	PFL2170c	7104	2600
	13	410301	411000	PF13_0048	2074	700
	13	723201	724600	PF13_0097	3822	1400
	13	1510401	1511400	PF13_0210	4825	1000
	13	1688001	1688800	PF13_0235	2577	800
	13	1689201	1691800	PF13_0235	6407	2600
	13	1692101	1693500	PF13_0235	3862	1400
	13	2579701	2580700	MAL13P1.322	3258	1000
	13	2580901	2581800	MAL13P1.322	2685	900
	13	2754601	2756200	MAL13P1.349	3835	1600
	13	2756501	2758000	MAL13P1.350	3721	1500
	13	2759701	2762600	MAL13P1.351	5624	2900
	13	2762701	2767800	MAL13P1.351	12111	5100
	13	2768001	2768500	MAL13P1.351	1090	500

(continued - OX005)

Amplifications	14	764201	766000	PF14_0179	4464	1800
	14	766201	770000	PF14_0179	7959	3800
	14	1156201	1159300	PF14_0273	7883	3100
	14	1323701	1325000	PF14_0315	3189	1300
	14	1326101	1328500	PF14_0315	7154	2400
	14	1389101	1390300	PF14_0326	4199	1200
	14	1670101	1670900	PF14_0389	3672	800
	14	2202501	2204800	PF14_0509	6294	2300
	14	2436701	2439400	PF14_0568	7645	2700
	14	2510001	2510600	PF14_0588	1669	600
	14	2511301	2511900	PF14_0588	1563	600
	14	2694801	2697500	PF14_0631	6338	2700

OX006	Chromosome	Begin	End	Gene name	Coverage	Size (in bp)
Deletions	7	612401	613300	MAL7P1.64	82	900
	8	434701	435800	PF08_0033	120	1100
	8	436301	437500	MAL8P1.38	102	1200
	8	443401	444100	PF08_0034	45	700
	8	461401	461900	PF08_0035	27	500
	8	462301	463400	PF08_0035	54	1100
Amplications	8	430401	431000	MAL8P1.36	2200	600
	8	453501	454800	MAL8P1.41	3703	1300
	8	455401	457100	MAL8P1.42	5851	1700

CNVs (larger than 500 bp) detected using the cn.MOPS

HB3	Chromosome	Begin	End	Gene name	Coverage	Size (in bp)
Deletions	4	615301	616300	PFD0660w	0	1000
	4	616901	618200	PFD0665c	4	1300
	7	612701	613400	MAL7P1.64	0	700
	8	430401	431000	MAL8P1.36	0	600
	8	436501	437400	MAL8P1.38	1	900
	9	1203001	1204900	PFI1475w	0	1900
	9	1205401	1206500	PFI1475w	12	1100
	14	3139301	3140900	PF14_0732	0	1600
	14	3142001	3144000	PF14_0733	0	2000
Amplifications	11	1934701	1935300	PF11_0503	1668	600

DD2	Chromosome	Begin	End	Gene name	Coverage	Size (in bp)
Deletions	1	515101	515700	PFA0650w	0	600
	2	104201	105200	PFB0100c	0	1000
	7	101601	102200	PF07_0007	0	600
	7	612701	613400	MAL7P1.64	1	700
	8	436501	437100	MAL8P1.38	8	600
	12	2168101	2169800	PFL2550w	0	1700
Amplifications	4	138901	139700	PFD0110w	1404	800
	5	888601	891700	PFE1095w	16394	3100
	5	892101	894800	PFE1095w	13710	2700
	5	896001	896600	PFE1100w	2469	600
	5	897401	899100	PFE1105c	9073	1700
	5	899201	899800	PFE1105c	3020	600
	5	901401	902200	PFE1110w	3722	800
	5	902601	903700	PFE1115c	4875	1100
	5	907801	918500	PFE1120w	57989	10700
	5	918601	922600	PFE1120w	21934	4000
	5	922801	928100	PFE1120w	28664	5300
	5	928401	936400	PFE1120w	42695	8000
	5	940201	941700	PFE1130w	7964	1500
	5	942701	943300	PFE1135w	2836	600
	5	944401	945000	PFE1140c	2839	600
	5	948901	951600	PFE1145w	14985	2700
	5	951901	953700	PFE1145w	9394	1800
	5	957801	962000	PFE1150w	22595	4200
	5	963201	964500	PFE1155c	6738	1300
	5	966101	969100	PFE1160w	16675	3000
	12	971301	972200	PFL1145w	4654	900
	12	972701	973400	PFL1150c	3984	700
	12	974301	975100	PFL1155w	4574	800

7G8	Chromosome	Begin	End	Gene name	Coverage	Size (in bp)
Deletions	1	532601	533200	PFA0665w	4	600
	3	871101	871700	PFC0915w	8	600
	4	283601	284200	PFD0260c	4	600
	7	612401	613400	MAL7P1.64	0	1000
	8	436501	437200	MAL8P1.38	5	700
	8	646801	647500	MAL8P1.70	22	700
	13	1895901	1896500	MAL13P1.236	4	600
	13	2445801	2446500	PF13_0323	8	700
Amplifications	5	374501	375300	PFE0440w	2256	800
	12	947401	958600	PFL1130c	46988	11200
	12	958801	960900	PFL1130c	9601	2100
	12	962701	966600	PFL1135c	14646	3900
	12	966901	967500	PFL1135c	2567	600
	12	969001	970100	PFL1140w	5091	1100
	12	971301	972200	PFL1145w	2897	900
	12	972701	973400	PFL1150c	2682	700
	12	974301	975100	PFL1155w	3439	800
	12	976801	977700	PFL1160c	2525	900

GB4	Chromosome	Begin	End	Gene name	Coverage	Size (in bp)
Deletions	1	515101	515700	PFA0650w	0	600
	3	122001	124800	PFC0110w	4	2800
	4	615301	616300	PFD0660w	0	1000
	4	616901	618200	PFD0665c	0	1300
	8	430401	431000	MAL8P1.36	0	600
	8	436501	437300	MAL8P1.38	0	800
Amplifications	4	139101	139700	PFD0110w	986	600
	5	374601	375300	PFE0440w	2007	700
	12	969101	969800	PFL1140w	2341	700
	12	971501	972100	PFL1145w	1759	600

OX005	Chromosome	Begin	End	Gene name	Coverage	Size (in bp)
Deletions	1	514001	514700	PFA0650w	11	700
	1	515001	515700	PFA0650w	21	700
	3	122001	124800	PFC0110w	7	2800
	4	615301	616300	PFD0660w	0	1000
	4	616901	618200	PFD0665c	0	1300
	4	988201	990500	PFD1030c	12	2300
	7	612701	613400	MAL7P1.64	9	700
	8	436501	437400	MAL8P1.38	0	900
	8	443101	443800	PF08_0034	6	700
Amplifications	1	527101	527800	PFA0665w	2444	700
	3	154101	154700	PFC0135c	1857	600
	3	306401	307000	PFC0285c	1634	600
	3	399001	399600	PFC0390w	2201	600
	3	481301	481900	PFC0475c	2184	600
	3	720201	720800	PFC0770c	1776	600
	3	757401	758200	PFC0810c	1996	800
	4	101401	102200	PFD0075w	2505	800
	4	104301	105100	PFD0080c	2345	800
	4	128001	129000	PFD0100c	3146	1000
	4	183801	184700	PFD0160w	2776	900
	4	296301	296900	PFD0265w	1956	600
	4	332501	333100	PFD0311w	2446	600
	4	390101	390900	PFD0385w	3026	800
	4	543601	544200	PFD0610w	2333	600
	4	649801	650800	PFD0669c	7961	1000
	4	897001	897600	PFD0970c	2227	600
	7	189801	190400	MAL7P1.17	1814	600
	7	449301	449900	MAL7P1.30	1744	600
	7	653301	654800	PF07_0056	6737	1500
	7	864301	864900	PF07_0081	3345	600
	7	866901	867600	PF07_0082	2784	700
	7	1152201	1153200	MAL7P1.149	2955	1000
	8	301301	303100	MAL8P1.19	12070	1800
	8	311901	312500	PF08_0019	2251	600
	8	579601	580300	MAL8P1.60	2030	700
	8	647401	648100	MAL8P1.70	2202	700
	8	664501	665200	MAL8P1.73	2500	700
	8	685601	686800	PF08_0060	3897	1200
	8	781101	781700	MAL8P1.92	1499	600
	8	860501	861200	PF08_0089	4006	700
	8	996401	997100	PF08_0101	2315	700
	8	1066101	1066700	MAL8P1.124	1689	600
	8	1140501	1141100	PF08_0116	1535	600
	8	1164201	1164800	MAL8P1.201	2366	600

(continued - OX005)

OX005	Chromosome	Begin	End	Gene name	Coverage	Size (in bp)
Amplifications	13	740601	741200	MAL13P1.92	2124	600
	13	1837701	1838800	MAL13P1.230	4359	1100
	13	1887401	1888500	PF13_0251	4240	1100
	13	1903301	1903900	MAL13P1.237a	1933	600
	13	2090801	2091400	MAL13P1.266	1645	600
	13	2144101	2144800	PF13_0279	2670	700
	13	2208901	2209500	MAL13P1.275	1861	600
	13	2323401	2324300	PF13_0314	2753	900
	13	2445401	2446100	PF13_0323	1858	700
	13	2579501	2580700	MAL13P1.322	3726	1200
	13	2650101	2650700	MAL13P1.333	1897	600
	13	2764801	2765800	MAL13P1.351	3360	1000
	14	1327401	1328300	PF14_0315	4035	900
	14	1670101	1670800	PF14_0389	3349	700
	14	2438701	2439300	PF14_0568	2458	600
	14	3035901	3036700	PF14_0710	2724	800

OX006	Chromosome	Begin	End	Gene name	Coverage	Size (in bp)
Deletions	8	436501	437100	MAL8P1.38	2	600
	13	1429401	1430000	MAL13P1.176	3	600
Amplifications	8	456001	456600	MAL8P1.42	2876	600
