# Supplementary material for: A Poisson hierarchical modelling approach to detecting copy number variation in sequence coverage data
Source: BMC Genomics. 2013 Feb 26;14:128. doi: 10.1186/1471-2164-14-128 (PMC3679970; doi:10.1186/1471-2164-14-128)
Supplement: Additional file 7 — Comparison between hits detected by the Poisson-Gamma model and the FREEC software. [file 1471-2164-14-128-S7.pdf]

Additional file 7

Comparison between hits produced by the Poisson-Gamma (PG) model using $\gamma = 99\%$ and the FREEC software.

The frequencies refer to the number of hits shared and exclusively detected by either methodology. Percentages are in relation to the overall number of deletions and amplifications identified by both methods, respectively. Hits detected exclusively by a given method were divided into two categories: one referring to hits in loci only identified by a given method and another referring to hits in loci targeted by both methods.

				Exclusive PG hits in				Exclusive FREEC hits in			
		Shared		Isolated loci		Shared loci		Isolated loci		Shared loci	
Type of CNV	Sample	n	%	n	%	n	%	n	%	n	%
Deletions	3D7	2	18.2	9	81.8	0	0.0	0	0.0	0	0.0
	HB3	181	55.5	141	43.0	0	0.0	3	0.9	3	0.9
	DD2	182	64.8	97	34.5	0	0.0	1	0.4	1	0.4
	7G8	91	29.7	152	49.7	0	0.0	41	13.4	22	7.2
	GB4	72	26.5	190	69.9	0	0.0	2	0.7	8	2.9
	OX005	60	48.9	148	45.3	0	0.0	9	3.8	10	4.2
	OX006	94	55.0	76	44.4	0	0.0	0	0.0	1	0.6
Amplifications	3D7	14	16.7	69	82.1	0	0.0	1	1.2	0	0.0
	HB3	20	8.0	226	90.8	0	0.0	2	0.8	1	0.4
	DD2	606	84.3	72	10.0	0	0.0	7	0.9	34	4.7
	7G8	228	39.0	115	19.7	0	0.0	151	25.8	91	15.6
	GB4	11	9.7	96	85.8	1	0.9	1	0.9	4	3.5
	OX005	206	9.7	813	37.5	0	0.0	453	20.9	694	32.0
	OX006	37	12.5	239	81.4	1	0.3	9	3.1	9	3.1

Comparison between hits produced by the Poisson-Gamma (PG) model ($\gamma = 99.9\%$) and the FREEC software.

				Exclusive PG hits in				Exclusive FREEC hits in			
		Shared		Isolated loci		Shared loci		Isolated loci		Shared loci	
Type of CNV	Sample	n	%	n	%	n	%	n	%	n	%
Deletions	3D7	2	18.2	9	81.8	0	0.0	0	0.0	0	0.0
	HB3	175	55.2	130	41.0	0	0.0	5	1.6	7	2.2
	DD2	175	63.9	90	32.8	0	0.0	3	1.1	6	2.2
	7G8	81	29.1	124	44.6	0	0.0	52	18.7	21	7.6
	GB4	72	27.4	181	68.8	0	0.0	2	0.8	8	3.0
	OX005	153	51.0	121	40.3	0	0.0	11	3.7	15	5.0
	OX006	93	55.0	73	43.2	1	0.6	1	0.6	1	0.6
Amplifications	3D7	14	77.8	3	16.7	0	0.0	1	5.6	0	0.0
	HB3	19	29.7	41	64.1	0	0.0	3	4.7	1	1.6
	DD2	586	84.3	48	6.9	0	0.0	24	3.5	37	5.3
	7G8	187	37.6	28	5.6	0	0.0	163	32.7	120	24.1
	GB4	6	10.5	41	71.9	0	0.0	1	1.8	9	15.8
	OX005	62	4.2	130	8.8	0	0.0	1033	69.7	258	17.4
	OX006	27	22.9	63	53.4	0	0.0	12	10.2	16	13.6
