# Supplementary material for: A Poisson hierarchical modelling approach to detecting copy number variation in sequence coverage data
Source: BMC Genomics. 2013 Feb 26;14:128. doi: 10.1186/1471-2164-14-128 (PMC3679970; doi:10.1186/1471-2164-14-128)
Supplement: Additional file 8 — Ternary diagrams plotting the joint proportions of shared and exclusively detected hits by the PG model, the FREEC software, and cn.MOPS. [file 1471-2164-14-128-S8.pdf]

Additional file 8

gamma=99%

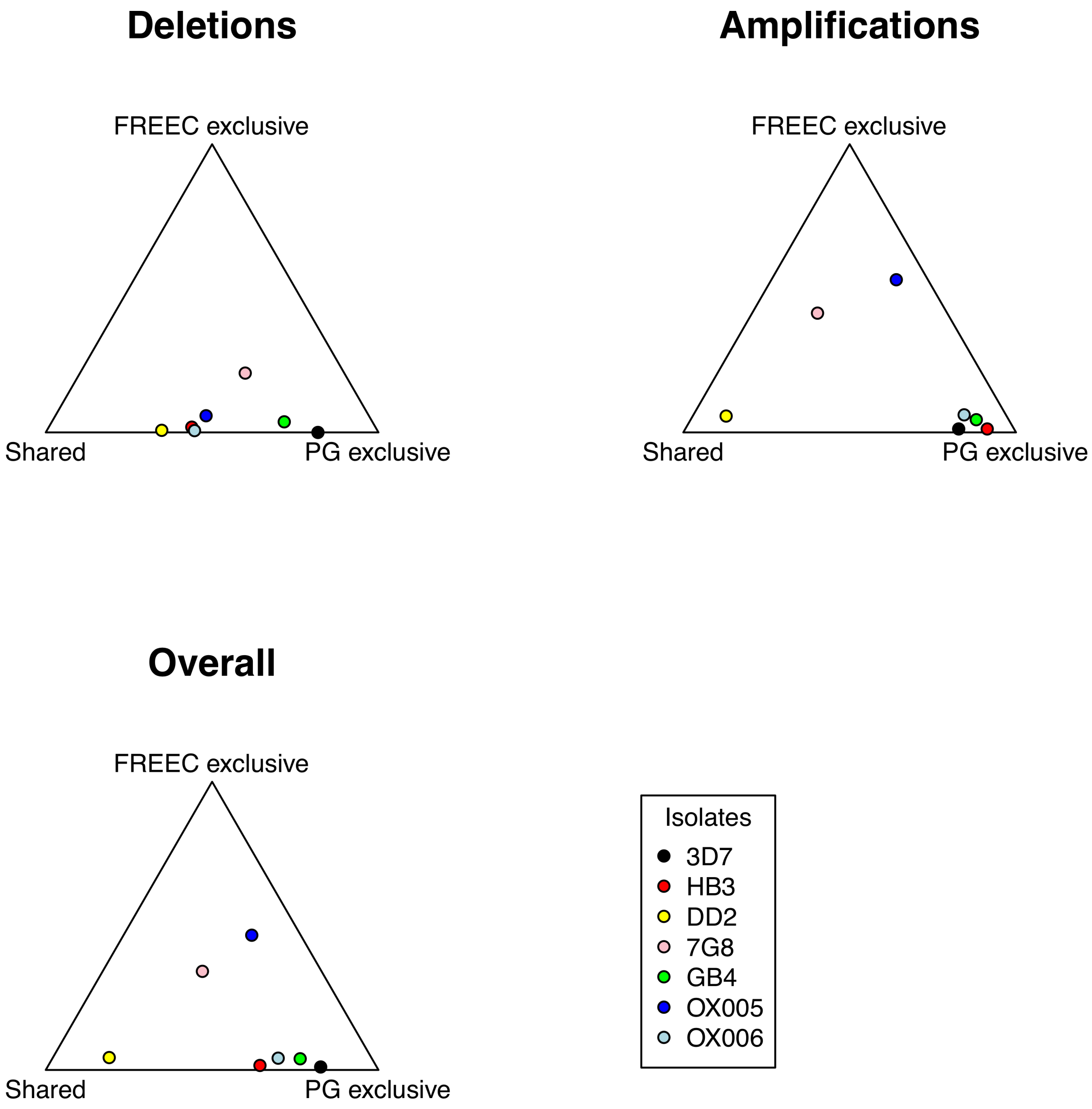

gamma =99.9%

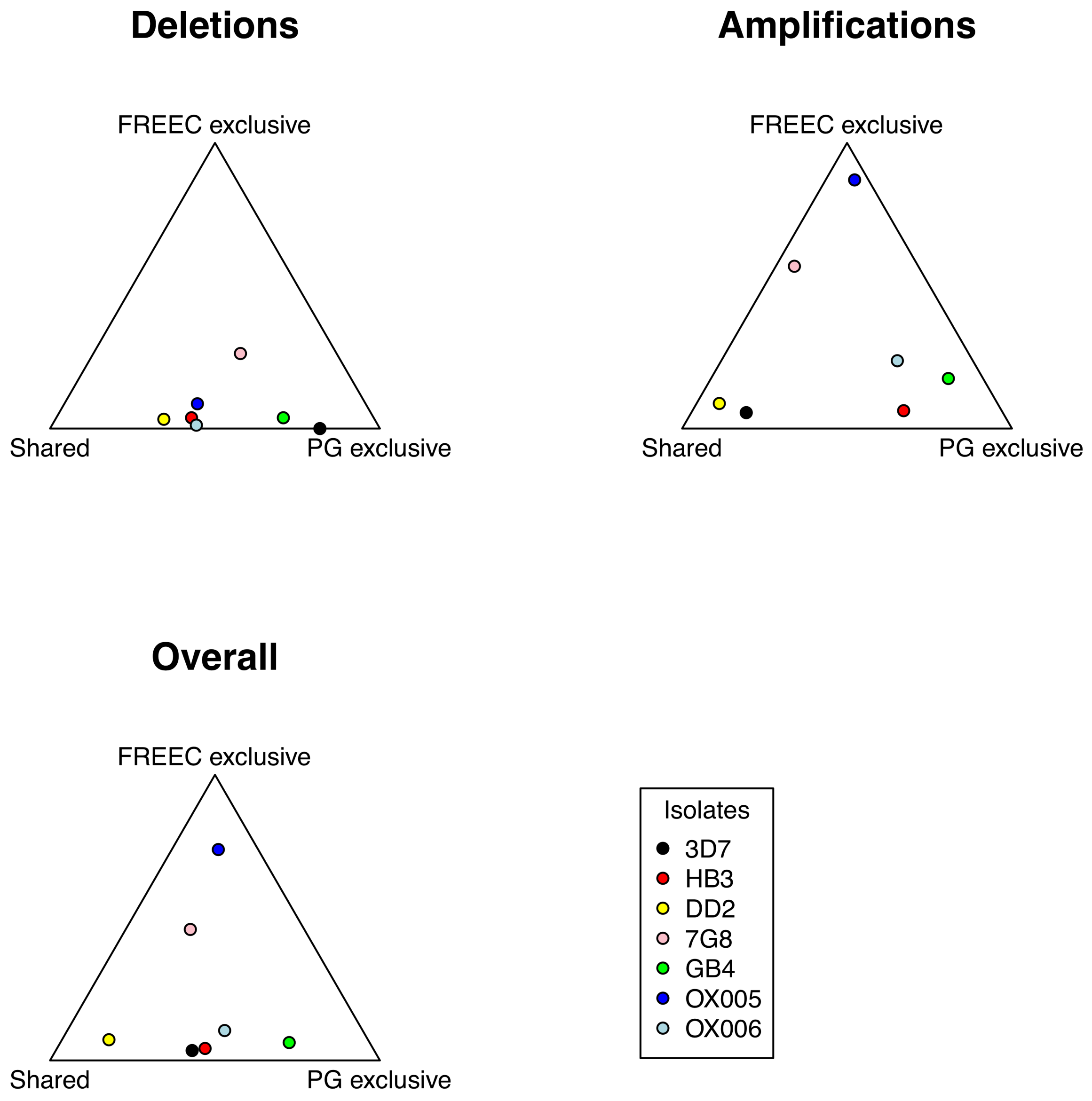
Deletions
FREEC exclusive
Shared
PG exclusive
Amplifications
FREEC exclusive
Shared
PG exclusive
Overall
FREEC exclusive
Shared
PG exclusive
Isolates
3D7
HB3
DD2
7G8
GB4
OX005
OX006

gamma=99%

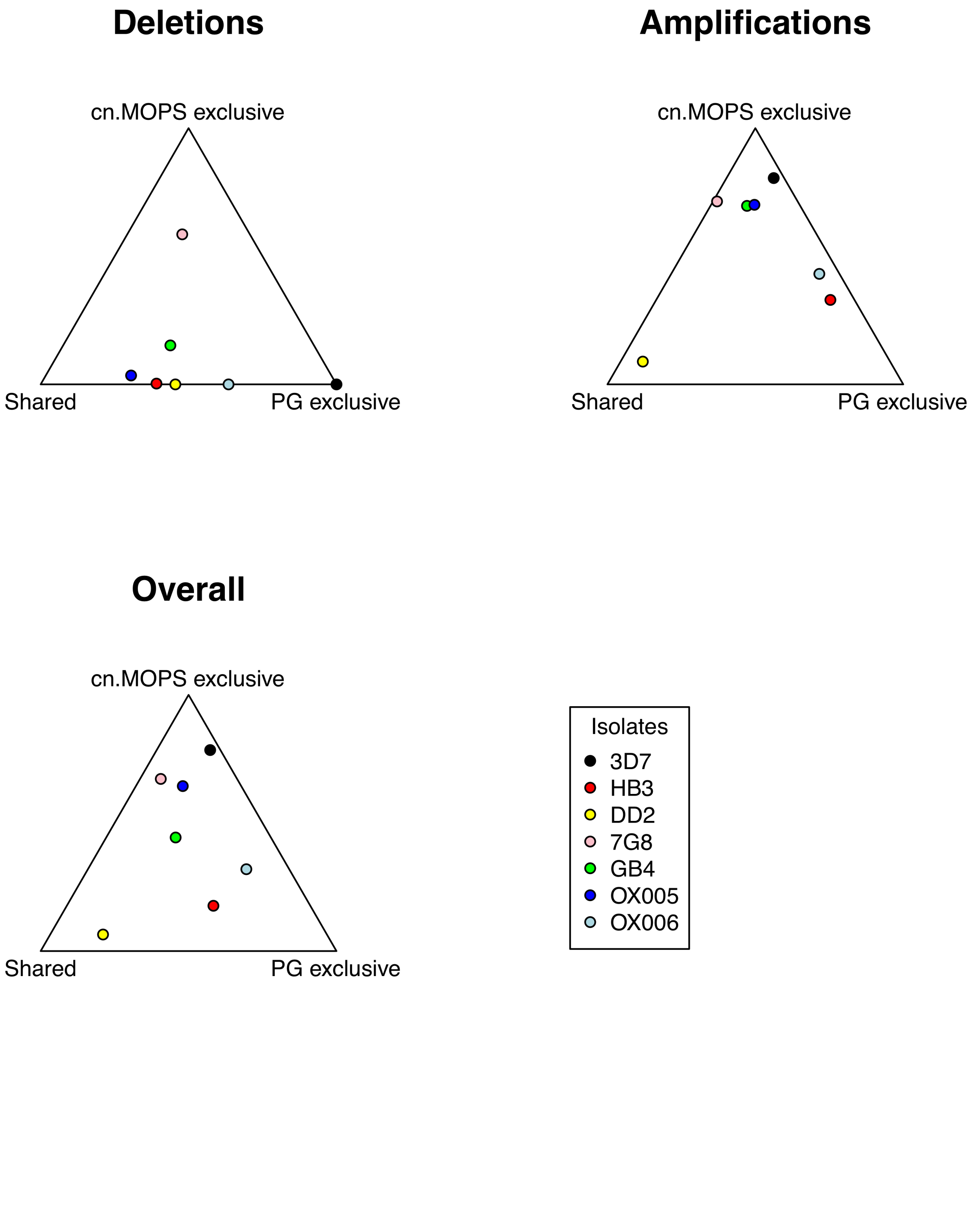

Deletions
cn.MOPS exclusive
Shared
PG exclusive
Amplifications
cn.MOPS exclusive
Shared
PG exclusive
Overall
cn.MOPS exclusive
Shared
PG exclusive
Isolates
3D7
HB3
DD2
7G8
GB4
OX005
OX006

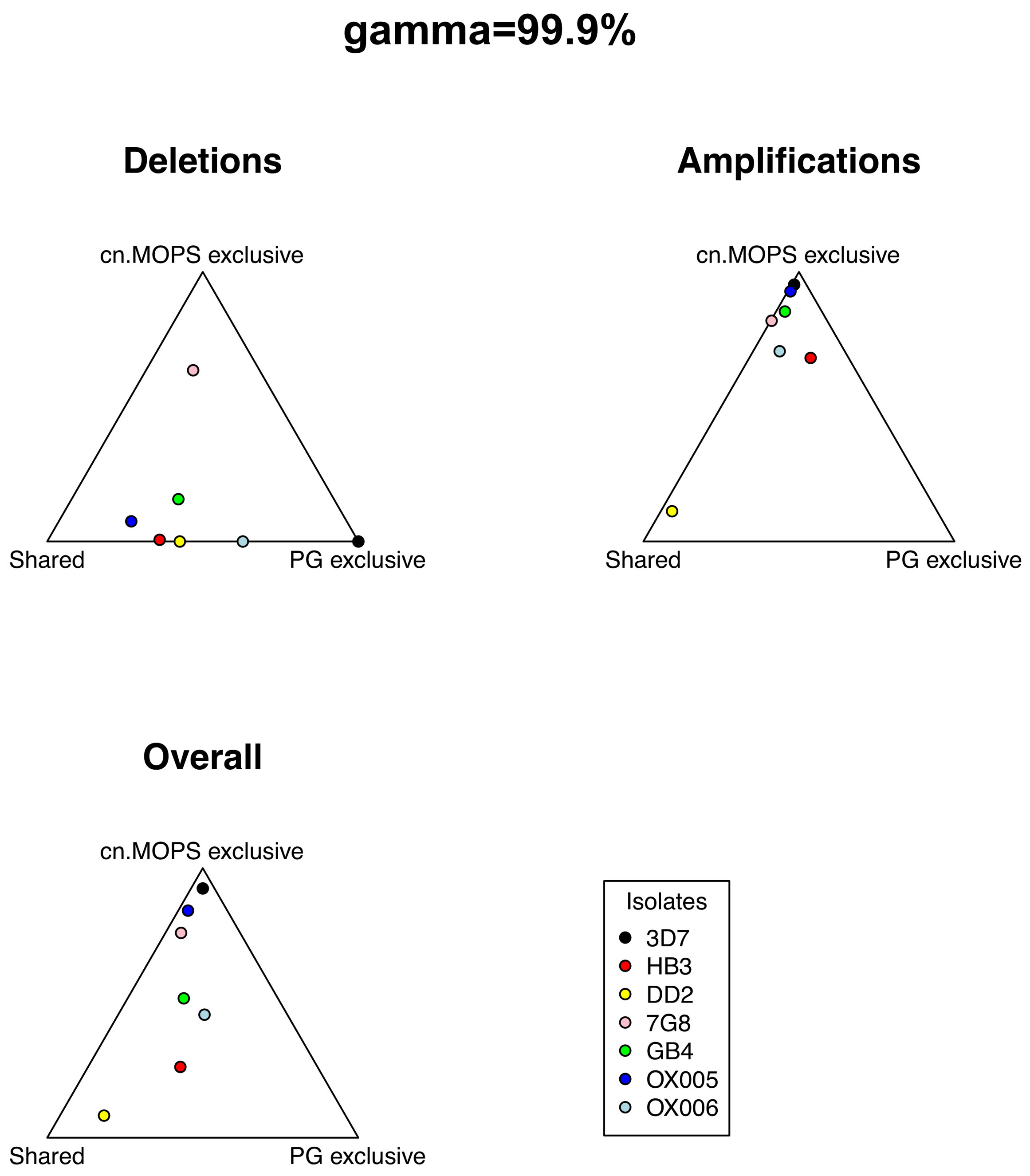
gamma=99.9%
Deletions
Amplifications
Overall
cn.MOPS exclusive
Shared
PG exclusive
Isolates
3D7
HB3
DD2
7G8
GB4
OX005
OX006
